# Combining Methods to Describe Important Marine Habitats for Top Predators: Application to Identify Biological Hotspots in Tropical Waters

**DOI:** 10.1371/journal.pone.0115057

**Published:** 2014-12-10

**Authors:** Laurie Thiers, Maite Louzao, Vincent Ridoux, Matthieu Le Corre, Sébastien Jaquemet, Henri Weimerskirch

**Affiliations:** 1 Centre d'Études Biologiques de Chizé, Station d'Ecologie de Chizé-La Rochelle UMR 7372, Centre National de la Recherche Scientifique, 79360, Villiers en Bois, France; 2 Instituto Español de Oceanografía, CO Xixón, Camín de l'Arbeyal s/n, 33212, Xixón, Spain; 3 Observatoire PELAGIS, CNRS, Université de La Rochelle UMS 3462, 5 allée de l'Océan, 17000, La Rochelle, France; 4 Université de la Réunion, Laboratoire ECOMAR (FRE 3560 CNRS), Avenue René Cassin, CS 92003 97744, Saint Denis Cedex 9, Ile de la Réunion, France; Ecole Normale Supérieure de Lyon, France

## Abstract

In tropical waters resources are usually scarce and patchy, and predatory species generally show specific adaptations for foraging. Tropical seabirds often forage in association with sub-surface predators that create feeding opportunities by bringing prey close to the surface, and the birds often aggregate in large multispecific flocks. Here we hypothesize that frigatebirds, a tropical seabird adapted to foraging with low energetic costs, could be a good predictor of the distribution of their associated predatory species, including other seabirds (e.g. boobies, terns) and subsurface predators (e.g., dolphins, tunas). To test this hypothesis, we compared distribution patterns of marine predators in the Mozambique Channel based on a long-term dataset of both vessel- and aerial surveys, as well as tracking data of frigatebirds. By developing species distribution models (SDMs), we identified key marine areas for tropical predators in relation to contemporaneous oceanographic features to investigate multi-species spatial overlap areas and identify predator hotspots in the Mozambique Channel. SDMs reasonably matched observed patterns and both static (e.g. bathymetry) and dynamic (e.g. Chlorophyll a concentration and sea surface temperature) factors were important explaining predator distribution patterns. We found that the distribution of frigatebirds included the distributions of the associated species. The central part of the channel appeared to be the best habitat for the four groups of species considered in this study (frigatebirds, brown terns, boobies and sub-surface predators).

## Introduction

Determining hotspots of biodiversity is common approach for setting conservation priorities [1]. Species belonging to the higher trophic levels play a key role in ecosystem functioning [2] and they are generally considered as good indicators of resources, especially in marine environments [3] Thus, the delineation of predator distribution is a suitable way of identifying hotspots, based on the assumption that predator assemblages will concentrate in areas where other species of lower trophic levels concentrate [4].

This is particularly the case for seabirds, which range over wide areas while concentrating for foraging on specific oceanographic features with enhanced availability of resources. Seabirds are easy to observe as they spend little time submerged [5] and are easy to monitor thanks to their land-based breeding, which facilitates monitoring of population abundance and the study of their at-sea distribution. Moreover, they are highly sensitive components of marine ecosystems and consequently major system shifts are reflected in their population dynamics [6]. So far, most studies using seabirds as indicators of biodiversity hotspots have been carried out in polar or temperate waters [7], [8], [9]. However, recent studies have begun to provide much needed knowledge from tropical regions [10], [11].

In tropical waters, resources are scarce and patchy compared to colder waters, so seabirds are not uniformly distributed in their environment [12] and many seabird species rely on sub-surface predators since these drive prey to the surface where seabirds can catch the prey more easily [13], [14]. Thus, interaction with sub-surface predators such as tuna and dolphins represent a major opportunity for seabirds to obtain food [12]. Some species are even near-obligate commensals of tunas [14]. This peculiar foraging strategy leads to large multispecific flocks of marine predators which are localised and ephemeral [13], [15].

Frigatebirds show extreme examples of this foraging strategy, since they never touch the sea surface, yet they rely entirely on marine resources [16]; they show this behaviour in both the tropical Pacific and Indian Oceans. Recent evidence highlights that frigatebirds associate to various oceanographic processes such as frontal zones between cyclonic and anticyclonic mesoscale eddies in the Mozambique Channel [16], [17] that will likely provide feeding opportunities. These highly dynamic frontal features are known to advect passive lower level organisms [18] and thus aggregate mobile predators [19], [20], [21], Based on this evidence, we hypothetised that frigatebird distribution mirrors other marine predator distribution in the Mozambique Channel, and the underlying physical oceanographic processes and prey distribution.

Several methods are used to determine marine predator distribution at sea. Vessel-based and aerial surveys are classical methods which have been used over decades to determine species distribution [22], [23], [24], [25], [26]. They present the advantage of allowing systematic survey over various temporal and spatial scales, but the disadvantage of being costly. More recently, biologging data have been used to determine the distribution of top predators [27]. In conservation studies, the use of tracking data or vessel-based surveys is widespread, and locations or densities of observations are commonly used to determine important areas for the animals. In addition, species distribution models using observational or telemetric data are useful tools to develop conservation plans [28], especially in environments that are not spatially and temporally homogenous.

The purpose of this study is to examine to what extent frigatebird distribution can be considered as a good indicator of diversity hotspots in tropical environments. For that, we developed species distribution models to identify key marine areas for tropical predators in relation to contemporaneous oceanographic conditions in the Mozambique Channel. In a first step, we assessed frigatebird distribution by combining data collected using various approaches, tracking technologies and classical aerial and at-sea surveys. Second, we compare the predictions of the distributions of frigatebirds and other seabirds typically constituting multispecific feeding flocks, such as sooty terns and boobies [29]. Third, we analysed the distribution of sub-surface predators such as tuna and dolphins in relation to seabird distribution [13], [14] to examine the spatial distribution of all four groups in the Mozambique Channel (frigatebirds, terns, boobies and sub-surface predators). For this purpose we determined the important marine areas for these taxa and created a map of high use areas from the outputs of the predictive models, allowing us to investigate the proportion of overlap between the distributions of the study taxa. Our study addressed the issue of determining if several sources of data and statistical processing are complementary in determining important areas at sea for top predators.

## Methods

### Predator distribution data

#### Ethics Statement

Ethical aspects of the study were approved by the *Préfet* of the *Terres Australes et Antarctiques Françaises* (TAAFs). All birds were handled and equipped in accordance with the ethics committee of TAAFs.

#### Tracking data

Tracking was carried out from Europa Island (22.3° S, 40.3° E), Mozambique Channel, SW Indian Ocean. Europa is the only breeding ground of frigatebirds (Great frigatebirds, *Fregata minor* and Lesser frigatebirds, *Fregata ariel*) in the Mozambique Channel [30]. Breeding frigatebirds were captured at nest and equipped with Platform Terminal Transmitters (PTT) during two seasons: 8 birds (Great frigatebirds - 2 males, 6 females) between August and October 2003 (see [16]) and 12 birds (6 Lesser frigatebirds – 2 males, 4 females - and 6 Great frigatebirds – 3 males, 3 females) between September 2011 and June 2012. Global Positioning System (GPS) loggers were also deployed on 10 birds (Great frigatebirds) between September and October 2008 (see [10]). Methods of attachment for PTTs and GPS were similar to those described in Weimerskirch et al [16]. The data for the two species and two sexes were pooled since there was no significant difference in range or distribution (unpublished data).

Tracking data were cleaned with an iterative backwards/forward speed filter routine by removing those unrealistic positions [31] with flight speeds higher than 65 km h^−1^ [32]. When tracking extended beyond the breeding period, foraging trips were divided between breeding and non-breeding periods. We considered a non-breeding trip when frigatebirds has no more central foraging behavior (i.e., no longer returning to the colony and moved to other areas). In order to obtain data with similar location frequency, GPS data for 2008 were randomly resampled at the mean frequency of ARGOS PTT, i.e. on average 1.57 h.

Kernel density distribution maps were generated from filtered locations of tracked frigatebirds using the kernelUD function from “adehabitat” package [33]. 95%, 75%, 50% and 25% density contours were represented for both breeding and non-breeding birds with a smoothing parameter of 0.5.

#### Vessel-based survey

Vessel-based surveys were conducted from 2002 to 2010 in different areas of the Mozambique Channel (more details in [34]). Seabirds and marine mammals were counted continuously along band transects at each side of the vessel, following Tasker (1984). For the surveys, 500 m wide bands were used instead of the traditional 300 m because of the lower bird density generally encountered in tropical waters. Distance from the vessel was estimated by using the radar of the vessel. Transects were then divided into 10 min bins with homogenous weather conditions and constant vessel speed (10 knots). See Jaquemet [34] for details of the method.

#### Aerial surveys

An aerial survey was conducted in December 2009 in the northern Mozambique Channel as part of the REMMOA survey [11]. Two aircraft with bubble windows were used to fly over previously defined, systematic line transects. The pilots aimed to maintain constant speed and altitude throughout the survey (90–95 knots, i.e. 167–176 km.h^−1^, and 183 m). One observer on each side used strip transects to count seabirds continuously in 500 m width bands, and marine mammals were counted over 200 m width bands as in Mannocci et al. [11].

### Environmental variables

Environmental variables were selected on the basis of their biological significance and the availability of data. Monthly composite environmental variables were downloaded from NOAA Coastwatch satellite (http://coastwatch.pfeg.noaa.gov/erddap/griddap/index.html) and aggregated on a 0.25° cell grid. We chose to use variables at a monthly scale instead of a finer temporal scale (e.g. weekly) to identify persistent areas, which could be used in memory-based foraging strategies [35]. Since frigatebirds are known from tracking studies to associate strongly with sub-mesoscale structures produced by mesoscale eddies [10], [16], [36], we tested the contribution of sea level anomalies to explain frigatebird distribution patterns at both weekly and monthly scales. Since these two temporal scales did not differ significantly in the obtained estimate values (Two-sample z-test, z = −1.45, p value = 0.148), a monthly scale was used for consistency between all variables tested in this study. Both dynamic variables, as Chlorophyll *a* concentration (Chloa, mg.m-^3^), sea surface temperature (SST, °C) and sea level anomaly (SLA, cm), and static ones as distance to the main colony (DCol, km) and bathymetry (Bathy, m) were retained as potential explicative variables of species distribution models (Table 1). We also included gradients of chlorophyll *a* concentration (Chloa_grad), SST (SST_grad), SLA (SLA_grad) and bathymetry (Bathy_grad), calculated with the slope function of the SDMTools package [37].

**Table 1:** Summary of the environmental variables for used for developing habitat models in these tropical waters, as well as their oceanographic interpretation.

Variables	Oceanographic interpretation	Source	URL
**Chloa (mg m-3)**	Proxy of primary production	MODIS	http://coastwatch.pfeg.noaa.gov/erddap/griddap/erdMHchlamday.html
**Chloa_grad**	Variability in Chloa distribution		
**SST (°C)**	Water mass distribution	MODIS	http://coastwatch.pfeg.noaa.gov/erddap/griddap/erdMHsstnmday.html
**SST_grad**	Proxy of water mass fronts		
**SLA (cm)**	Geostrophic structures (Eddies)	AVISO	http://atoll-motu.aviso.oceanobs.com/?action=listproductmetadata&service=AvisoDT&product=dataset-duacs-dt-upd-global-merged-msla-h.html
**SLA_grad**	Eddies fronts		
**Bathy (m)**	Coastal and pelagic habitats	ETOPO	http://coastwatch.pfeg.noaa.gov/erddap/griddap/etopo180.html
**Bathy_grad**	Topographic features		
**Dcol (km)**	Distance to the colony		

Dynamic variables included Chlorophyll *a* concentration (Chloa), sea surface temperature (SST), sea level anomaly (SLA) and their spatial gradients (grad).

Static variables included distance to the colony (DCol), bathymetry (Bathy) and its spatial gradient.

### Species distribution models

Species distribution models (SDMs) were developed separately for frigatebirds according to observational method (tracking data and vessel-based surveys). In addition, SDMs were also developed for associated seabird species (e.g., brown terns, boobies) and sub-surface predators (e.g., delphinidae and tunas).

#### Data processing

To compare SDM outputs, we first needed to standardise disparate datasets such as tracking, vessel- and aerial-based surveys (Louzao et al 2009). We selected the spatial resolution based on the coarsest scale of all datasets (corresponding to 0.25° of sea level anomaly). We therefore constructed a standard grid covering the Mozambique Channel (limited to the spatial extent of tracking and vessel-based surveys) with a spatial scale of 0.25°. Predator and environmental data were aggregated over this standard grid with a temporal resolution of 1 month.

For tracking data of breeding birds, filtered locations of each foraging trip were assigned on corresponding cells of the standard grid and time spent per unit area was used to define presence cells. Since these types of data provide only presence records, we generated pseudo-absence data for frigatebird tracks. For each foraging trip, we estimated the number of presence cells and we generated an equal number of pseudo-absences in cells that were not crossed by the bird with a limit of 1000 km from the colony (i.e., according to the mean maximum range of breeding birds).

For vessel-based surveys, we used only data collected between September and December to match the temporal coverage of frigatebird tracking data which corresponds to the incubation and early chick rearing period on Europa [38]. For the focal species, frigatebirds, observations were composed of both Great *Fregata minor* and Lesser *Fregata ariel* frigatebirds. The brown tern community in the Mozambique Channel is composed mainly of sooty terns [34]. The two different species of boobies considered here were the red-footed booby (*Sula sula*) and the masked booby (*Sula dactylatra*), the former being the most abundant in the Mozambique Channel. Delphinidae and tunas are well known for their common feeding ecology and the presumed association of foraging frigatebirds with those sub-surface predators [14], [29]. As a consequence, we pooled all taxa of sub-surface predators in one unique group: individuals of the genera *Stenella*, *Tursiops*, *Delphinus* and *Peponocephala*, as well as tuna schools.

Seabird and marine mammal numbers were aggregated over the grid with standard 0.25° cell, according to month and year. For visualisation purposes, we estimated predator density according to the observation effort in each 0.25° cell (number of animals per km^2^). Densities were re-coded into a binary presence/absence variable, indicative of whether at least 1 individual was recorded within a given 0.25° cell. For aerial surveys, we applied the same procedure for the unique month of sampling.

Finally, contemporaneous environmental variables were then extracted for presence and absence data according to month and year.

#### Model development

Species distribution models were developed within the Information Theoretic approach to identify those environmental variables that better describe distribution patterns of tropical predators [39], [40], [41], [42]. To understand the presence and absence of predators, we developed logistic regressions. We used Generalized Linear Mixed Models (GLMMs) to account for individual or campaign effects by incorporating a random term in the models. Models were fitted with a binomial distribution using the glmmML function from the glmmML package within the R environment [37]. For tracking data, SDMs were developed for breeding birds only.

Prior to modelling, environmental variables were normalized (i.e., a mean of 0 and an SD of 1 due to differing ranges of variables) to allow comparison of their relative influence on SDMs. Co-linearity between environmental variables was checked using Spearman rank correlation. For each pair of correlated variables (r>0.6), only the most informative one was kept for analysis (*i.e.,* the variable with the lowest Akaike Information Criteria, AIC, value). Based on this preliminary screening, we removed Chloa_grad in the case of tracking data and vessel-based surveys, except for sub-surface predators for which we removed Chloa and not considered DCol. In addition, we performed an exploratory analysis with univariate models to consider the importance of linear and non-linear (quadratic) effects of environmental variables on the presence and absence of predators. Neither types of effect differed significantly in the AIC value of the model (less than 2 points), we therefore chose to include the simplest effects in further analyses.

#### Model selection strategy

Models were built for all possible linear combination of ‘non-correlated’ explanatory variables and no interaction terms were included. The resultant models were then ranked according to their AIC value. In addition, we calculated the Akaike weight (*A_w_*) for each model, which represents the relative likelihood of candidate models [43]. When the *A_w_* of the model with lowest AIC was below 0.90, a model averaging procedure was used to account for model uncertainty [44]. To obtain average models, we first identified the 95% confidence set of models when the cumulative sum of *A_w_* values was >0.95. Then averaged estimates were calculated using the estimates of the 95% confidence set of model weighted by their A_w_ [44]. Linear predictors were calculated using intercept and coefficients of the average models, and the presence probability (*P_r_*) was calculated following Louzao et al. [42]: 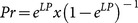



#### Model evaluation

The model evaluation phase is a crucial step in estimating the predictive performance of SDMs to ensure that model predictions are transferable and generalizable [45]. We used the area under the curve (AUC) to evaluate the predictive performance of SDMs (AUC >0.9, excellent; from 0.9 to 0.8, good; from 0.8 to 0.7, moderate; from 0.7 to 0.6, poor; and from 0.6 to 0.50, unsuccessful) [46]. In addition, there is wide consensus that SDMs should be validated using independent data sets to avoid over-fitting data or over-rating model performance [45]. We applied a cross-validation procedure to assess the predictive performance of the averaged model resulting from the Information-Theoretic Approach using independent test datasets. Models within the 95% confidence set were fitted to 70% randomly selected observations on the test dataset and tested on the remaining 30% estimating the AUC value in both cases. This process was repeated 1000 times and the predictive performance of predators models were assessed based on AUC scores (mean, upper and lower 95% confidence interval) (McAlpine et al. 2008). The averaged SDMs were transferable and generalizable when the lower 95% CI limit did not include the 0.5 value. Thus, we could conclude that SDMs predictions could be used to predict beyond training dataset and draw reliable predictions on species distribution.

In the case of tracking without DCol term, the independent dataset was composed by non-breeding frigatebird tracking data from 2011. Whether both breeding and non-breeding distribution provided similar modelling outputs, we could assume that both population components exploit similar foraging habitats. Regarding vessel-based surveys, we built the 95% confidence set of models with data from 2002, 2005 and 2007 and the independent dataset was composed by data from 2008 and 2009 surveys.

#### Spatial patterns of predictions

We mapped the predicted spatial distribution of frigatebirds (tracking data {TD} and vessel-based surveys {VBS}), terns (VBS), boobies (VBS) and sub-surface predator (VBS). Dynamic environmental variables were extracted yearly for each month (September-December) from 2002 to 2011 covering the study temporal window. Then, predictions were obtained by applying the 95% confidence set of models to predict presence probability of tropical predators. The 10-years predictions were averaged for each month and the standard deviation (SD) was used as an index of habitat stability (low and high SD representing stable and unstable habitats, respectively) [41].

### Spatial overlap of tropical predators

We were interested in analysing the spatial overlap of all four taxa in the Mozambique Channel. For this purpose we determined the important marine areas for these taxa to produce a map of High Use Area using vessel-based predictive models. For each average model, we estimated the ROC parameters (Receiver Operator Curve) of sensibility (percentage of presences correctly predicted) and specificity (percentage of absences correctly predicted) [47]. Then, we estimated the probability threshold (*P_th_*) at which sensibility and specificity were maximised (S1 Table). Cells with values higher than *P_th_* were considered as suitable, whereas the opposite identified unsuitable cells. For each year of the study period, continuous surface probabilities from the seasonal mean (September to December) were recoded into the binary output of suitable and non-suitable to identify key marine areas for each species. We summed up for how many species a cell was suitable developing an overlap index ranging from 0 (suitable for 0 taxa) to 4 (suitable for all taxa). Finally, a map of this average suitability index was generated. Using this overlap index, we investigated the proportion of overlap between the distributions of the four study groups. Spatial overlap was calculated for each pair of taxa by dividing cells where both taxa were present by the sum of cells where these taxa were present following [48], for each year of the study period.

## Results

### The spatial distribution of frigatebirds

**Figure 1:**
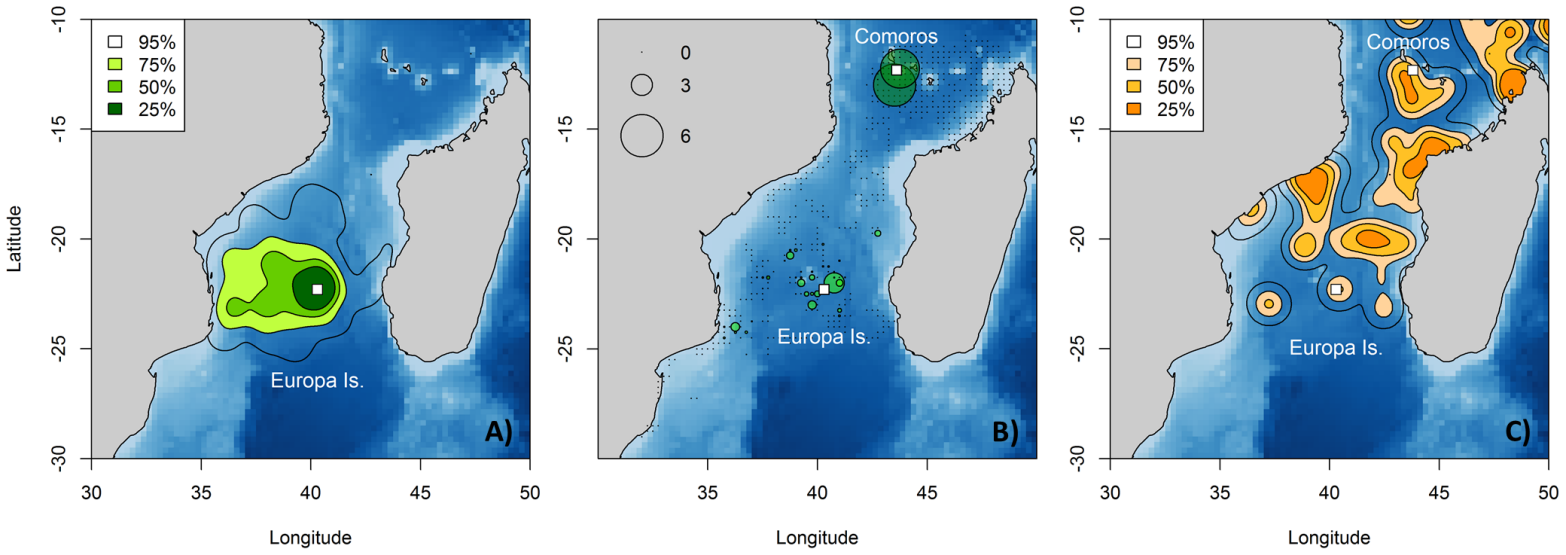
Frigatebird distribution in the Mozambique Channel collected from different methods. Kernel density contours from analysis of ARGOS locations of (a) breeding frigatebirds during October 2003, 2011 and 2008 and (c) non-breeding frigatebirds during October 2011. White rectangles represent the breeding colony in Europa Island and the wintering ground in the Comoros. Ninety-five percent, 75%, 50% and 25% contours are represented. (b) Density (number of birds.km^-2^) of frigatebirds observations from vessel-based surveys between September and December all years confounded (light green) and from aerial survey in December 2009 (dark green). Zeros represent areas surveyed where no individuals were recorded.

Frigatebirds used most of the Mozambique Channel (MC) with two main concentration areas: around Europa Island and Comoros (S1 Figure). Breeding frigatebirds mainly concentrated around Europa where they nest with a westward extension of their foraging range shown by both tracking and vessel-based surveys (Fig. 1a and 1b). Although non-breeding frigatebirds dispersed widely in the Mozambique Channel they tended to favour coastal areas both along Madagascar and Mozambique (Fig.1c). In the northern MC, the marine areas around the Comoros archipelago were used heavily, as indicated by the higher abundances seen on the aerial surveys (Fig. 1b).

Traditionally the distribution of central place foragers has been modeled including distance to the colony as explanatory variable but because of its masking effect on the others selected variables when included in our models (S2 Table), distance to the colony was removed in the tracking-based model.

**Figure 2:**
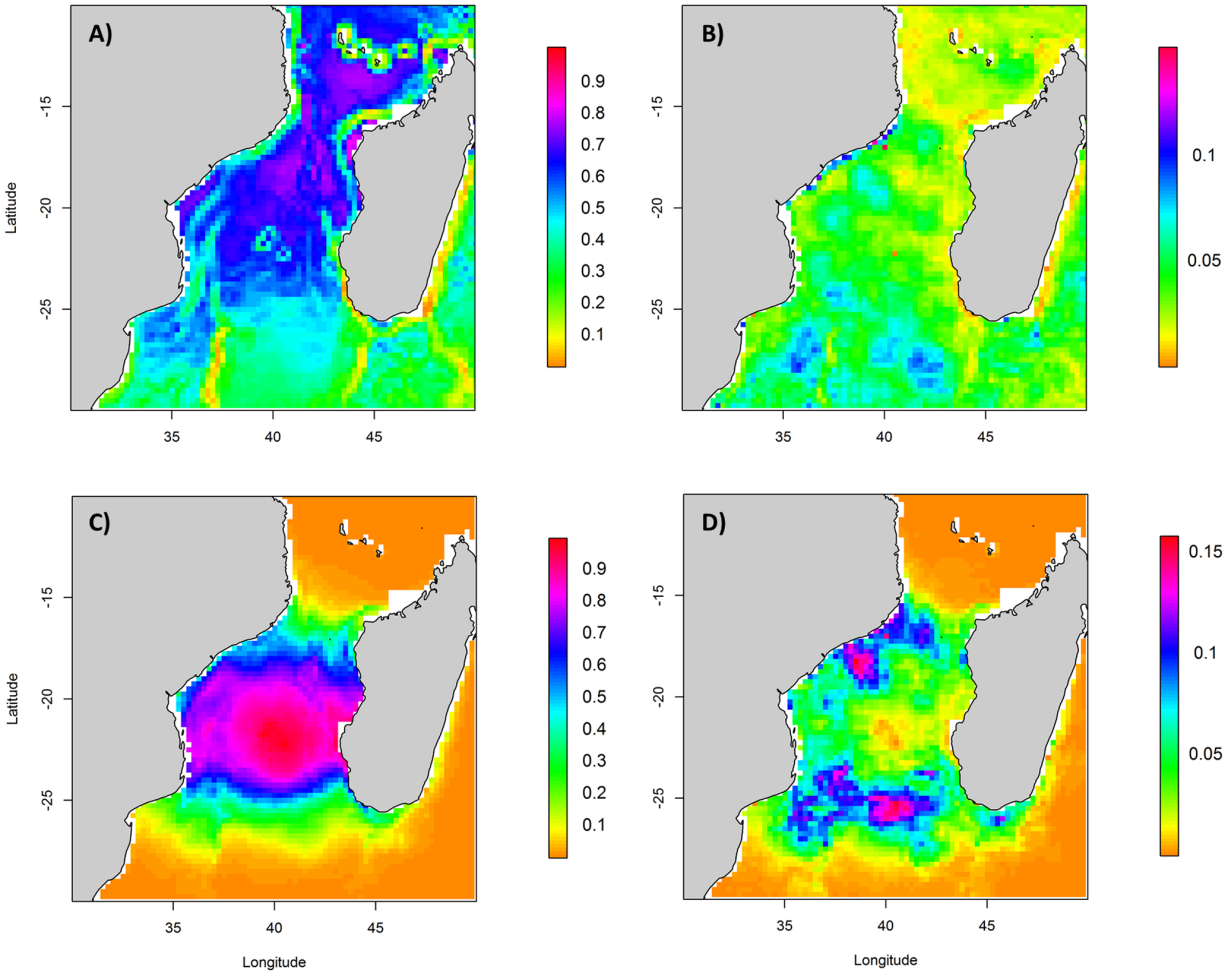
Predictive spatial models of breeding frigatebird distribution in the Mozambique Channel during the 2002–2010 period. Mean presence probability in October based on tracking data (a) and vessel-based surveys (c) and (b, d) associated uncertainty maps (represented by the standard deviation of monthly predictions).

Regarding modelling output, a model averaging approach was selected over the best single model approach since the model with the lowest AIC value showed an A_w_ value of 0.73. The 95% confidence set of models included 2 models (S3 Table). The average model showed a reasonable predictive performance of 0.68. The main environmental variables driving frigatebird distribution patterns, by order of importance (averaged values), were Chloa, SST and Bathy_grad, with Chloa and Bathy_grad having a negative effect (Table 2). Suitable habitat for frigatebirds is thus characterized by oceanic zones of the northern Mozambique Channel. Cross-validation procedures for the selected model provided high AUC values both for the training and the tested dataset suggesting that this model could be applied generally. The highest probabilities of presence occurred in the central sector of the MC (north of Europa) and the Comoros (Fig. 2a). Variability of predictions from year to year was extremely low (max: 0.13), with highest values in the southern part of the study area (Fig. 2b).

**Table 2:** Summary of the outputs of the average models and C-index cross-validation values.

Studied taxa		Frigatebird	Frigatebird	Boobies	Terns	Sub-surface predators
**Data collection approach**		Tracking	Vessel	Vessel	Vessel	Vessel
**Parameters**						
	**INT**	0.24±0.11	−2.79±0.36	−2.27±0.24	1.43±0.16	−1.84±0.21
	**Chloa**	−0.8±0.79	−0.59±0.47	0.32±0.11	0.16±0.08	-
	**SST**	0.61±0.36	0.53±0.28	0.43±0.19	0.13±0.09	0.52±0.27
	**SLA**	−0.22±0.08	−0.31±0.14	−0.01±0.01	0.01±0.11	−0.11±0.04
	**Bathy**	0.3±0.1	1.11±1.20	0.32±0.11	−0.84±0.88	0.06±0.03
	**DCol**	-	−2.89±8.64	−1.27±1.65	−0.97±1.14	-
	**Chloa_grad**	-	-	-	-	0.12±0.03
	**SST_grad**	−0.02±0.03	−0.26±0.11	0.27±0.08	−0.01±0.11	−0.22±0.08
	**SLA_grad**	0±0	0.1±0.03	−0.51±0.3	0.04±0.09	−0.5±0.31
	**Bathy_grad**	−0.54±0.03	0.12±0.05	0.3±0.1	0.78±0.58	−0.4±0.22
**AUC average model**		0.68	0.83	0.78	0.79	0.70
**Cross-validation**						
	**AUC _training_**	0.80±0.01	0.85±0.02	0.84±0.05	0.79±0.04	0.65±0.04
	**AUC_Test_**	0.79±0.02	0.57±0.07	0.68±0.06	0.77±0.04	0.65±0.13

Values are mean ± SD. INT: intercept. AUC: Area Under the Curve. For the other abbreviations see legend of Table 1.

Regarding the low A_w_ value of the better model for frigatebirds developed using vessel-based observations (0.2), we used a model averaging approach with 36 models in the 95% confidence set (S4 Table). The resulting average model included Chloa, SST, SLA, DCol, Bathy, SST_grad, SLA_grad and Bathy_grad. DCol and Bathy were the variables with the main influence, with DCol having a negative effect (Table 2). The AUC value of this model was 0.83, with cross-validation value of training dataset 0.85±0.02 and test dataset 0.57±0.07. The highest presence probabilities concentrated around Europa Island (Fig. 2c). Model showed low uncertainty (0.15), at the boundaries of the high presence probability area (Fig. 2d). With the aim of making the two model outputs comparable, we also developed a vessel-based model without DCol (S2c Figure, S5 Table). The AUC value of the model was poor (0.52). Driving parameters were SST and Chloa, Chloa having a negative effect (S5 Table). The highest probability occurs between Europa and the Comoros, in the central part of the channel. Uncertainty was relatively low (0.12) and principally located in the middle part of the Mozambique Channel (S2d Figure).

### The distribution of associated seabirds

**Figure 3:**
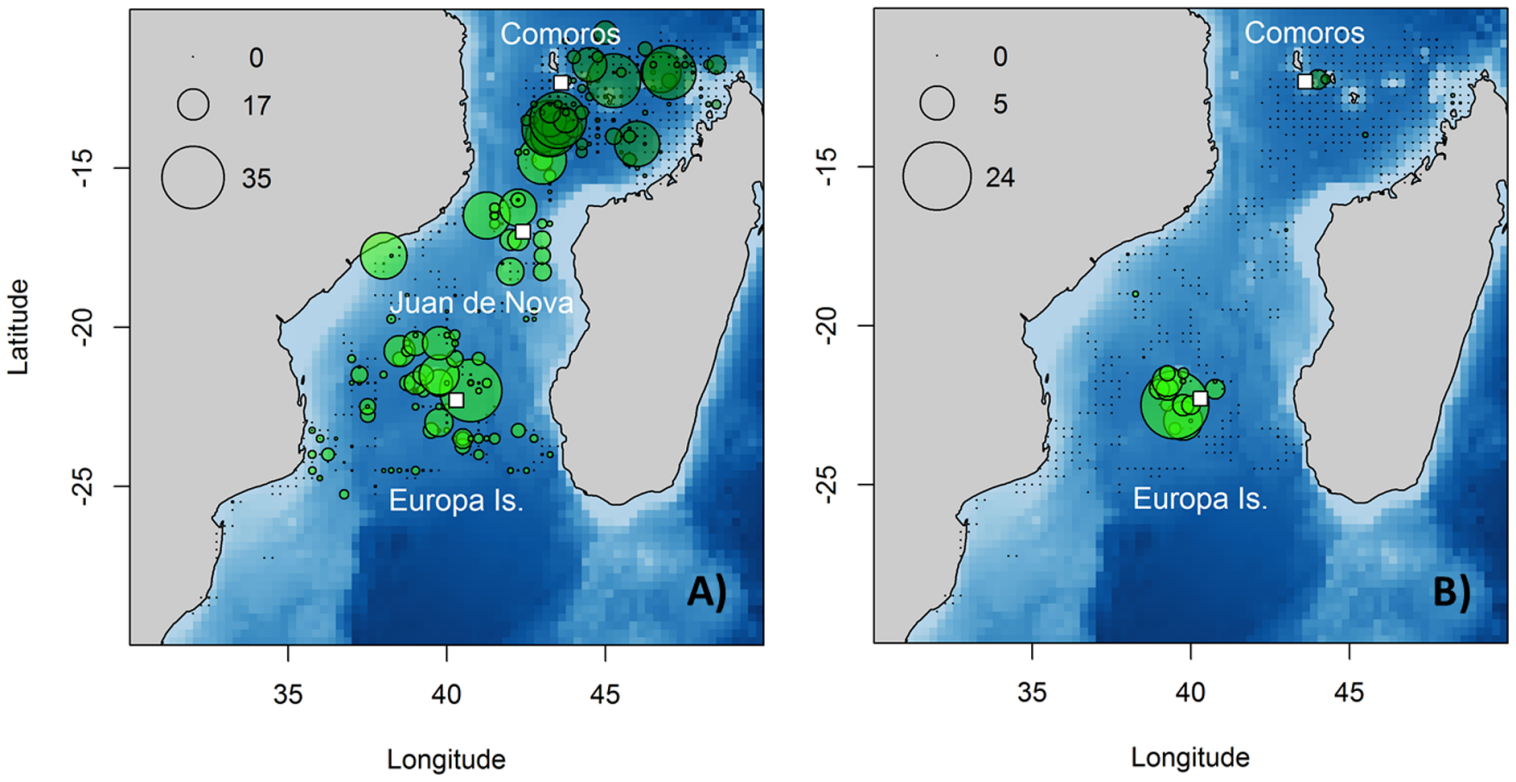
Associated seabird descriptive spatial analysis in the Mozambique Channel. (a) Tern and (b) boobies density (number of birds.km^−2^) from vessel-based surveys between September and December (2002–2010) (light green) and from aerial survey in December 2009 (dark green) are represented. White rectangles represent the breeding colony in Europa Island, Juan de Nova and the wintering ground in the Comoros.

Based on vessel surveys, terns showed a wide distribution in the MC with their main concentration area in the south-west of the Channel (more than 2000 birds in a single observation) followed by an important concentration area in the north-east sector (Fig. 3a). Aerial observations confirm the wide distribution of sooty terns in the surveyed area, but show larger aggregations around the Comoro archipelago and very low densities on the continental shelf of Madagascar (Fig. 3b).

**Figure 4:**
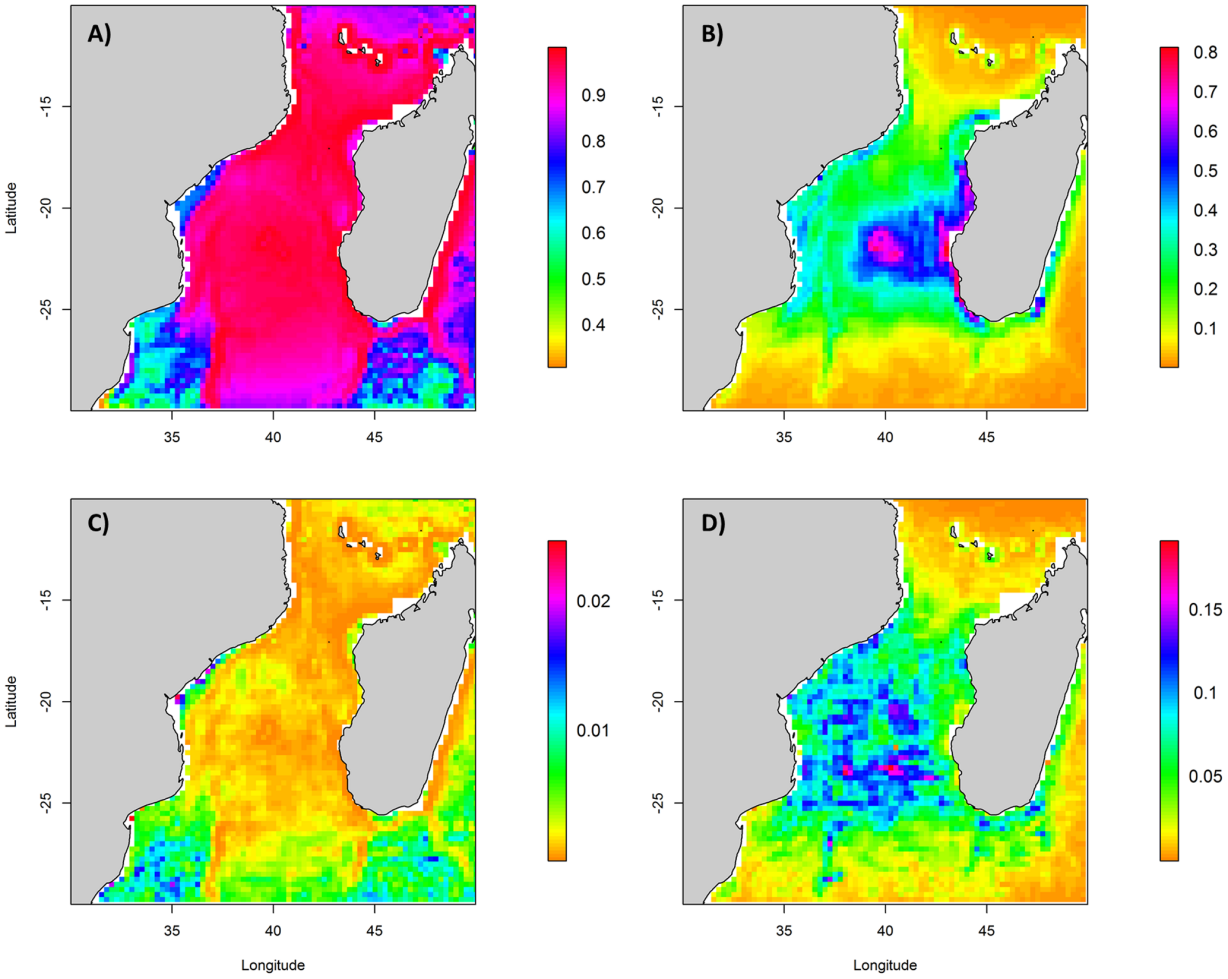
Output of species distribution models for terns and boobies from vessel-based observations in October (2002–2010) along the Mozambique Channel. Climatology of mean presence probability from (a) terns and (c) boobies and (b, d) associated uncertainty map (SD of monthly predictions).

Since the A_w_ value of the best model was only 0.48 (S6 Table), an average model was calculated within the 12 models of the 95% confidence set. This model was mainly driven by DCol, Bathy and Bathy_grad (Table 2), with Dcol and Bathy exhibiting a negative effect in accordance with the descriptive results of the surveys. The probability of presence of terns was mainly associated with static features, and was higher close to the colonies, in shallow waters with high bathymetric gradients. In spatial terms, high probabilities of presence occurred in all offshore waters of the MC, with lower probabilities in coastal areas (Fig. 4a). The predictive performance of this model was good. The average model (AUC: 0.79) showed very low uncertainty values, lower than 0.02 (Fig. 4b). Cross-validation scores were 0.79±0.04 and 0.77±0.04 for training and test dataset respectively.

Vessel-based observations of boobies showed a distribution pattern concentrated around Europa (Fig. 3b). No other individuals were observed in the rest of the surveyed area. Aerial observations of boobies were scarce too. They showed an aggregation around the Comoros and some individuals between the Comoros and the northwest coast of Madagascar.

Since the model with the lowest AIC exhibited an A_w_ of 0.1 (S7 Table), a model averaging approach was used, based on the 66 models of the 95% confidence set. The main variable influencing the boobies average model was DCol followed by dynamic variables such as SLA_grad and SST (Table 2). Thus, the probability of presence of the boobies was higher close to the main colony (Europa). Within the dynamic landscape of the Mozambique Channel they associated negatively to eddies fronts and positively with warm waters. The average model (AUC: 0.78) predicted high presence probabilities around Europa Island and along the western coast of Madagascar where bathymetric gradients are high (Bathy_grad influenced presence positively for boobies) (Fig. 4c). Maximum uncertainties (max. 0.16) were located in the South-western part of the Mozambique Channel (Fig 4d). Cross-validation values were 0.84±0.05 for training and 0.68±0.06 for test data.

These model outputs showed that terns and boobies share similar habitats with frigatebirds, supporting the hypothesis of feeding association. Moreover, raw data from boat-based surveys showed that, when feeding, frigatebirds occurred with boobies and terns, in 20% and 86% of observed flocks respectively, finally, in 18% of the feeding events of frigatebirds, they were associated with both boobies and terns.

### The distribution of sub-surface predators

**Figure 5:**
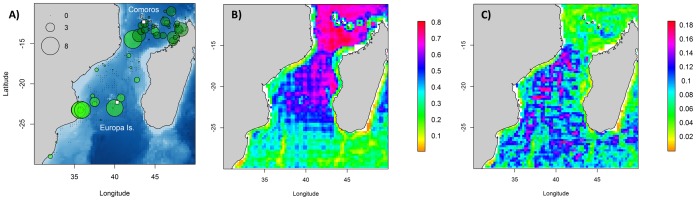
Sub-surface predators descriptive spatial analysis represented by (a) density (number of individuals.km^−2^) from vessel-based surveys between September and December (2002–2010) (light green) and aerial survey in December 2009 (dark green) in December 2009. Output of species distribution models for sub-surface predators represented by (b) mean probability of presence for October and (c) its associated uncertainty map (SD of presence probability) based on vessel-based observations between 2002 and 2010.

Vessel-based surveys recorded sub-surface predators around Europa and along the coast of Mozambique (Fig. 5a), whereas aerial surveys recorded high densities between the Comoros archipelago and the northwest coast of Madagascar (Fig. 5b).

Regarding modelling output, a model averaging approach was used since the model with the lowest AIC value showed an A_w_ value of 0.19. The 95% confidence set of models included 57 models (S8 Table). The average model showed a reasonable predictive performance AUC value of 0.70, and was mainly driven by SST, SLA_grad and Bathy_grad, SST having a positive effect (Table 2). Thus, sub-surface predators were associated with warm waters and negatively to fronts. In spatial terms, maximum values occurred in the northern MC and, to a lesser extent, in the middle part of the Channel (Fig. 5b), corresponding to the area of the Channel where SST is high and less dynamic. The average model is generalizable to other conditions since the AUC values of cross-validation scores did not include 0.5 (AUC for training: 0.65±0.04; AUC for test: 0.65±0.13).

### The marine predator community in the Mozambique Channel

Using thresholds at which sensibility and specificity calculated for each average model developed were maximised (S1 Table), cells showing predicted probability over this threshold were classified as suitable while cells below the threshold were considered unsuitable. Combining suitability maps for the four groups of species studied, which were calculated for each year of the study (2002–2011) during the breeding months (September to December), a map of the important areas for the predators was designed. Then, an overlap index was estimated between pairs of taxa which show a mean overlap value ranging from 0.26 to 0.41 (Table 3). Among the three groups of seabirds, the highest overlap value is found in pairs of taxa including frigatebirds, showing that this species is the most likely to cover the habitats of the other species in its distribution.

**Table 3:** Proportion of species overlap calculated for each year of the study period.

Species	Frigatebirds	Terns	Boobies
**Frigatebirds**	NA	NA	NA
**Terns**	0.29±0.05	NA	NA
**Boobies**	0.41±0.02	0.26±0.06	NA
**Sub-surface predators**	0.31±0.03	0.35±0.04	0.26±0.05

Values are mean ± SD.

**Figure 6:**
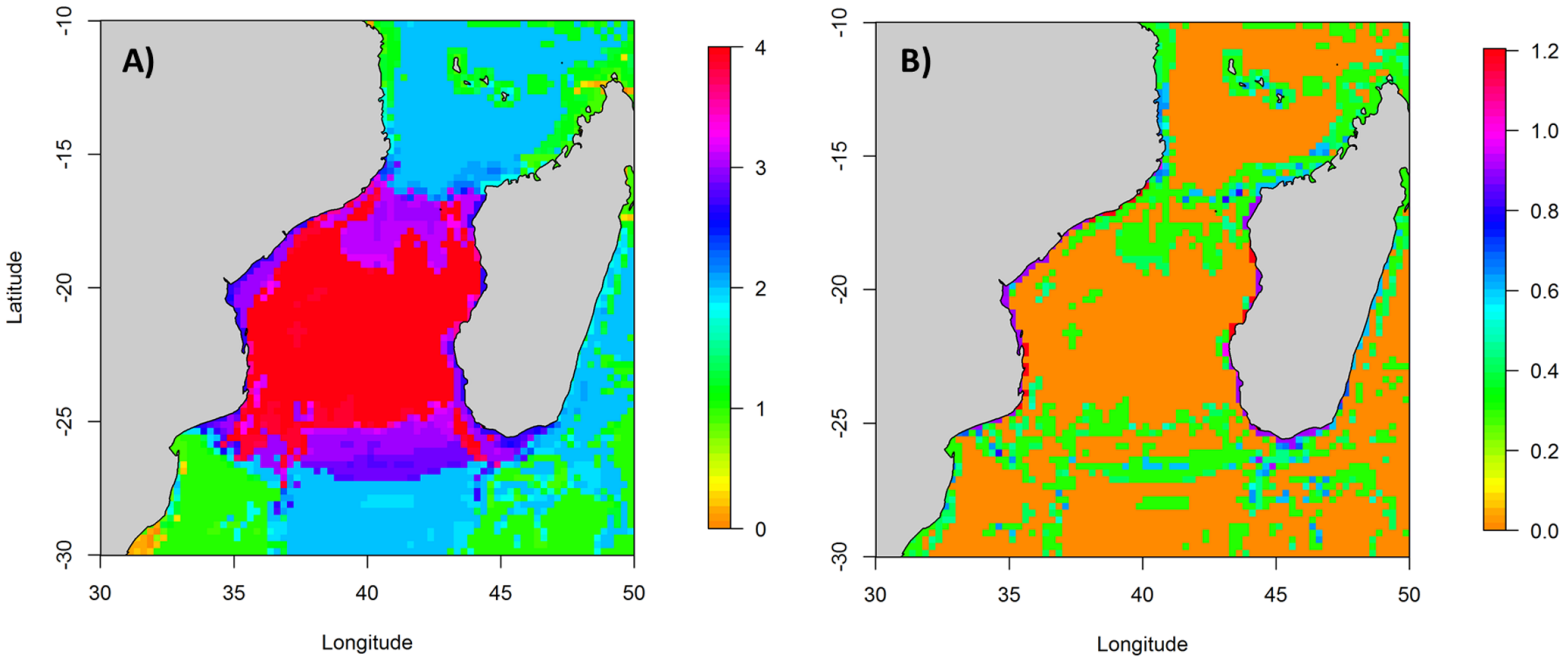
Map of the Mean High Use areas based on (a) vessel-based spatial predictions for frigatebirds, terns, boobies and sub-surface predators species in the Mozambique Channel, and (b) associated uncertainty.

Moreover, the output of the map of the important areas showed that suitable areas for all groups of birds and mammals tend to congregate. Cells predicted as suitable for all species were concentrated in the central and southern MC (Fig. 6a), with coastal areas along both Madagascar and Mozambique being less suitable than offshore waters. Finally, the lowest values occurred in the southern part of the study area. Uncertainties are very low (Fig. 6b) and show that overlap areas are persistent over years.

## Discussion

Distribution data from both tracking and at-sea observations are complementary, and these sources of information are nowadays increasingly combined in conservation studies [42], [49], [50]. Tracking data provide accurate information on individuals of known provenance, age and breeding status whereas at-sea observations allow large-scale investigation of the distribution pattern of all population components (i.e., juveniles, immatures, non-breeders and breeders). At-sea observations also inform on biological interactions, feeding behaviour and feeding events, which can only be speculated with tracking devices. In this study, distribution data of several species of seabirds and marine mammals obtained by a combination of data from three platforms were compared, with the ultimate aim of determining important areas for these marine predators.

### Marine habitats of frigatebirds in the Mozambique Channel

By combining two different but complementary approaches, tracking and vessel or aerial observations, we were able to show that two different components of the population have distinct distributions. Although breeding and non-breeding birds share similar foraging areas near Europa and its westward extension, densities were higher for breeders around Europa (observed from tracking and vessel) and for non-breeders in the northern sector of the Mozambique Chanel (as indicated by tracking and aerial surveys). While breeders forage as central place foragers around Europa, non-breeding adults move to the Comoros area to exploit different marine areas, using roosting sites as a central place for foraging [51].

During breeding, birds perform foraging trips ranging around 1000 km to feed on flying fish and squids, which are the prey boobies and terns feed on as well [52]. However, there is evidence for differences in the diet of frigatebirds according to their breeding status and age class. Isotopic signatures of non-breeding birds exhibit higher variability and overall lower trophic levels in their prey, showing a shift in frigatebirds diet when they are no longer central place foragers.

The distribution of frigatebirds is negatively influenced by Chlo *a*, suggesting that the birds do not seem to track high primary production areas in the Mozambique Channel. However, this result should be interpreted with caution as frigatebirds do not feed on primary producers. A natural delay between phytoplankton development and presence of micronekton and associated fish species is likely to occur, especially when satellite imagery is used at a monthly scale. Successions of eddies moving southward in the Mozambique Channel mix water masses to create a dynamic ecosystem [53] where it is difficult to determine how seabirds respond to ephemeral biotic structures. Moreover, high chlorophyll a concentration is associated with Zambezi river mouth in the MC. The turbidity of this area may also explain the negative relationship observed.

### Spatial overlap of frigatebirds and other species

Terns are widely distributed in the Mozambique Channel, and breed in extremely large numbers on Europa, Juan de Nova and the Glorioso archipelago [38], [54]. Both vessel-based and aerial surveys revealed the wide oceanic distribution of brown terns (represented mainly by sooty terns) throughout the MC. In particular, vessel-based observations show concentrations in the western part of the MC, where high densities of frigatebirds occur, suggesting the existence of shared foraging areas where they likely feed in common flocks in offshore waters [14], [34].

In the MC, the only large colony of boobies (red-footed boobies) is located on Europa [15]. Boobies showed a distribution exclusively driven by the location of their colony. This is revealed by both at-sea observations and tracking data that indicate relative short ranges of breeding adults red-footed boobies from Europa. It is consistent with previous studies which showed directly, using tracking or at-sea observations, that breeding red footed boobies from Europa forage close to their colony to a maximum of 150 km from the island [34], [55]. Other booby species also have relatively short ranges compared to frigatebirds, especially brown boobies and masked boobies [56], [57], these latter species being present in small numbers in the MC. This is also in accordance with wide-ranging surveys indicating that boobies occur mainly in coastal flocks [14], and show no dispersal of the non-breeding part of the population [55]. The range of frigatebirds in the MC encompasses that of boobies, in agreement with the results of an isotopic study showing that frigatebirds and boobies share the same feeding area and overlap with terns [52].

Sub-surface predators were widely distributed in the MC, with a higher density in the northern part (Fig. 5a). Our model predicted their distribution in central and northern MC which is consistent with the distribution of purse seine fishing effort for tunas in the MC [36]. This distribution overlaps widely with those of the seabirds, which is in accordance with the well-known role of dolphins and tunas in aggregating multispecific feeding flocks in the Mozambique Channel [14].

Despite the dynamic nature of the environmental parameters used here, all models presented satisfying predictive performance, and AUC values ranging from 0.68 to 0.83 with moderate to good cross-validation values (Table 2). Some areas emerge as potential zones of importance for all the top predators.

### Marine predator hotspots

Marine predator hotspots were identified in offshore waters of the southern MC, represented by a longitudinal ellipsoide between the mouth of Zambezi River to the west and the coast of Madagascar to the east, including Europa Island (Fig. 6 a).

The same species shown to form multispecific feeding flocks in previous studies [14], [34] were found to co-occur in this study. As a consequence, the species that share similar foraging areas are likely to be vulnerable to a shift in the density or distribution of any species that aggregate their prey or signals the presence of prey aggregations. In particular, any depletion of tuna species, which are targeted by industrial fisheries, would be of great concern.

In the case of seabirds, our results show a clear influence of the colony, in particular for frigatebirds and boobies. Breeding seabirds are central place foragers and need to return to the colony to incubate, brood or feed their chicks [58], [59]. Thus, their foraging trips are limited in time and distance from their nest. This foraging behaviour is particularly evident in the case of tracking data from breeding frigatebirds equipped at Europa Island. However, the influence of the distance to the colony on seabird distribution is also highlighted in at-sea observations from both vessel-based and aerial surveys, although both breeding and non-breeding birds are involved. Indeed, Europa Island is a major breeding site for numerous species of seabirds [30], the only breeding site for frigatebirds in the MC, and distance to the colony has been shown to be an important predictor of their distribution [60]. The neighbourhood of the colony or a roosting site could also be an important factor in the distribution of non-breeding birds, especially since frigatebirds are the only seabirds that never rest on the surface of the sea.

Besides the location of main colonies, the marine habitats shared by study species are characterised by high SST and Bathy values, and negative Bathy_grad and Chloa. These oceanographic conditions describe oceanic waters of the Mozambique Channel, characterised with low chlorophyll *a* levels and warm water. In particular, the western oceanic sector of the MC appears to be an area where top predators concentrate, particularly frigatebirds, terns and dolphins. This area of high mesoscale activity, with several eddies flowing southward year long, is already known to be attractive for frigatebirds that forage at the edges of the eddies [10], [16]. Indeed, these oceanic processes enhance primary production, and concentrate micronekton at the periphery of eddies [61].

The negative relation observed between the presence of predators and Chlo *a* could be linked to the absence of foraging in coastal areas, where highest values of Chlo *a* occurred, particularly on the mouth of the Zambesi River, off Mozambique.

### Limitations of the study and future directions

The accuracy of the predicted presence areas is constrained by both the spatial and the temporal scales of the study. Indeed, monthly composite environmental data cannot reflect fine scale dynamic parameters such as upwelling filaments (cool, elevated-chlorophyll a waters) that have been shown to drive the distribution of frigatebirds [10], [62]. In the same way, the use of monthly scale variables did not necessarily reflect temporal integration between the detection of primary production, and peak prey availability for top predators. However, using weekly data of SLA does not improve the performance of the model, which indicates that given the highly dynamic variability of these processes, defining the ideal temporal scale for habitat modelling in the MC is difficult. In addition, there is as yet no information on the time lag between the presence of high levels of chlorophyll *a* and a peak of prey in the Mozambique Channel.

Identifying foraging areas instead of presence areas for top predators in the Mozambique Channel might have improved the addressing of preliminary hypothesis, however, models using foraging information were not successful (results not shown), because altitude data that indicate when frigatebirds are foraging [16] would be necessary to determine precisely when birds are feeding. In addition raw observations from this study show that foraging frigatebirds and boobies were often associated with foraging terns, and frequently with sub-surface predators.

## Conclusions

Tracking data on breeding birds can give a biased view of the distribution of a species if there are differences between breeding and non-breeding birds. Because frigatebirds are wide ranging species (>500 km), we found a good agreement between the distributions of breeders and non-breeders from tracking data and observations at sea.

Overall, habitat models do not predict precise hotspots apart from the vicinity of Europa, suggesting that although frigatebirds concentrate at sub-mesoscale features associated with the edge of eddies, because these habitats are very mobile over short periods of time, they are not predictable at a monthly scale. This underlines again the high unpredictability of tropical waters, and the difficulty of identifying precisely important zones at fine temporal and spatial scales.

Nevertheless, data from different sources in this study showed consistent results. Mapping high use areas reveals a wide overlap between the four groups of species studied here, suggesting that the distribution of frigatebirds is a good indicator of top predator concentrations at large and meso-scales.

## Supporting Information

S1 Figure Map of the frigatebird trips collected from September to October 2003, September to October 2008 and September to December 2011. White rectangles represent the breeding colony in Europa Island and the wintering ground in the Comoros.
(TIF)

S2 Figure Output of species distribution models for frigatebirds including DCol. Climatology of mean presence probability (a) from frigatebirds in October (based on tracking data from 2003 and 2011) and (c) vessel-based observations and (b, d) associated uncertainty map (standard deviation of monthly predictions).
(TIF)

S1 Table Threshold of maximised sensibility and specificity for vessel-based models.
(DOC)

S2 Table Ranked set of best candidates frigatebirds tracking model and average model integrating distance to the colony (DCol). Corrected Akaike Information Criterion (AICc), measure of each model AIC relative to the best one (d) and Akaike Weight (w) are presented. Values are mean ± SD.
(DOC)

S3 Table Ranked set of best candidates frigatebirds tracking model and average model. Corrected Akaike Information Criterion (AICc), measure of each model AIC relative to the best one (d) and Akaike Weight (w) are presented. Values are mean ± SD.
(DOC)

S4 Table Ranked set of best candidates frigatebirds at-sea observations model and average model. Corrected Akaike Information Criterion (AICc), measure of each model AIC relative to the best one (d) and Akaike Weight (w) are presented. Values are mean ± SD.
(DOC)

S5 Table Ranked set of best candidates frigatebirds at-sea observations model and average model. Corrected Akaike Information Criterion (AICc), measure of each model AIC relative to the best one (d) and Akaike Weight (w) are presented. Values are mean ± SD.
(DOC)

S6 Table Ranked set of best candidates terns at-sea observations model and average model. Corrected Akaike Information Criterion (AICc), measure of each model AIC relative to the best one (d) and Akaike Weight (w) are presented. Values are mean ± SD.
(DOC)

S7 Table Ranked set of best candidates boobies at-sea observations model and average model. Corrected Akaike Information Criterion (AICc), measure of each model AIC relative to the best one (d) and Akaike Weight (w) are presented. Values are mean ± SD.
(DOC)

S8 Table Ranked set of best candidates sub-surface predators at-sea observations model and average model. Corrected Akaike Information Criterion (AICc), measure of each model AIC relative to the best one (d) and Akaike Weight (w) are presented. Values are mean ± SD.
(DOC)
