# Supplementary figures and images for: Combining Methods to Describe Important Marine Habitats for Top Predators: Application to Identify Biological Hotspots in Tropical Waters
Source: PLoS One. 2014 Dec 10;9(12):e115057. doi: 10.1371/journal.pone.0115057 (PMC4262456; doi:10.1371/journal.pone.0115057)

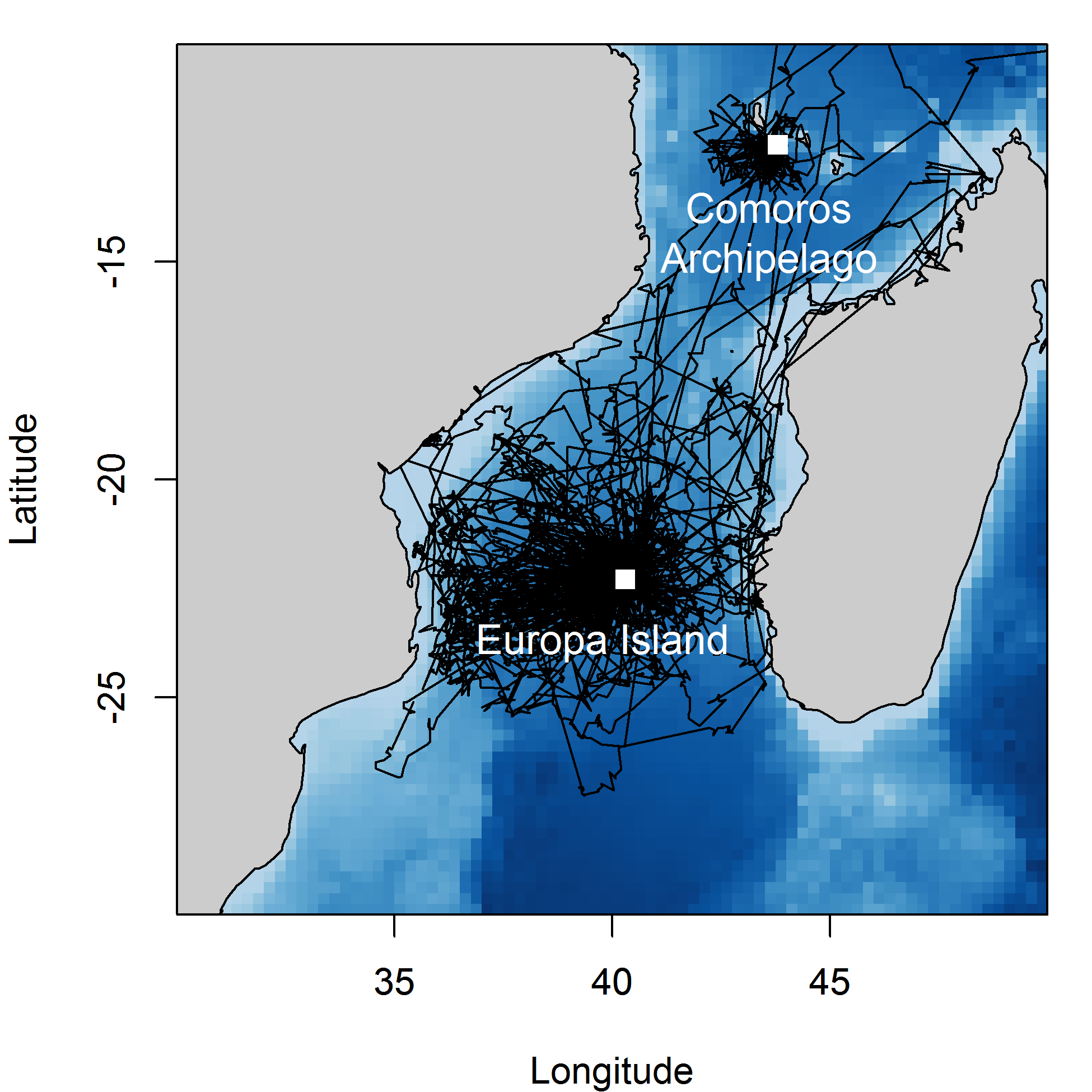

Supplement: S1 Figure — Map of the frigatebird trips collected from September to October 2003, September to October 2008 and September to December 2011. White rectangles represent the breeding colony in Europa Island and the wintering ground in the Comoros. (TIF) [file pone.0115057.s001.tif]

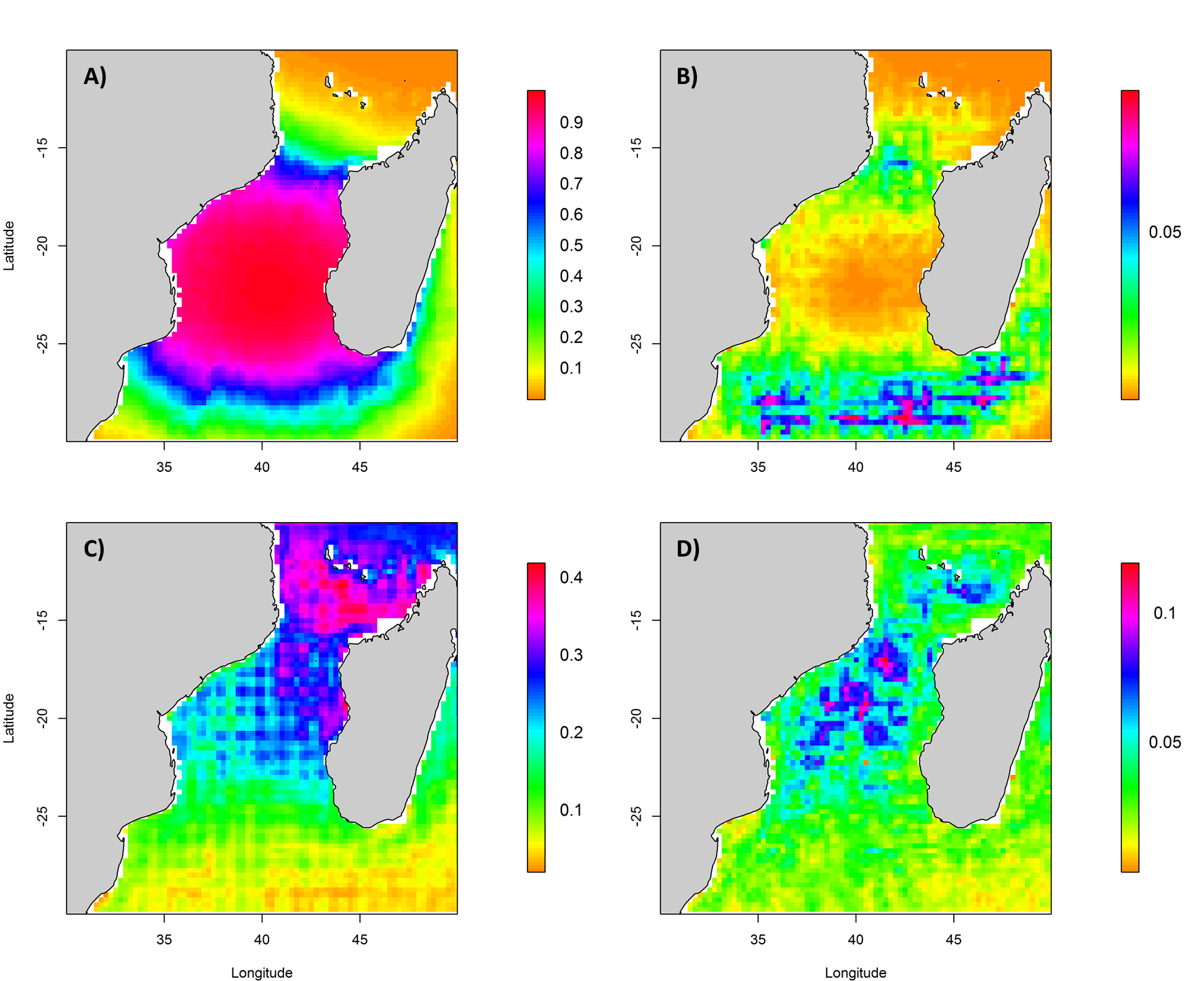

Supplement: S2 Figure — Output of species distribution models for frigatebirds including DCol. Climatology of mean presence probability (a) from frigatebirds in October (based on tracking data from 2003 and 2011) and (c) vessel-based observations and (b, d) associated uncertainty map (standard deviation of monthly predictions). (TIF) [file pone.0115057.s002.tif]
