# Supplementary material for: Combining Methods to Describe Important Marine Habitats for Top Predators: Application to Identify Biological Hotspots in Tropical Waters
Source: PLoS One. 2014 Dec 10;9(12):e115057. doi: 10.1371/journal.pone.0115057 (PMC4262456; doi:10.1371/journal.pone.0115057)
Supplement: S1 Table — Threshold of maximised sensibility and specificity for vessel-based models. (DOC) [file pone.0115057.s003.doc]

| **Model** | **Threshold** | **Sensibility** | **Specificity** |
| --- | --- | --- | --- |
| Frigtebirds | 0.20 | 0.73 | 0.74 |
| Terns | 0.80 | 0.69 | 0.70 |
| Boobies | 0.16 | 0.70 | 0.69 |
| Sub-surface predators | 0.15 | 0.67 | 0.62 |
