# Supplementary material for: Combining Methods to Describe Important Marine Habitats for Top Predators: Application to Identify Biological Hotspots in Tropical Waters
Source: PLoS One. 2014 Dec 10;9(12):e115057. doi: 10.1371/journal.pone.0115057 (PMC4262456; doi:10.1371/journal.pone.0115057)
Supplement: S3 Table — Ranked set of best candidates frigatebirds tracking model and average model. Corrected Akaike Information Criterion (AICc), measure of each model AIC relative to the best one (d) and Akaike Weight (w) are presented. Values are mean ± SD. (DOC) [file pone.0115057.s005.doc]

| Model | INT | Chloa | SST | SLA | Bathy | SST_grad | SLA_  grad | Bathy_  grad | AICc | d | w |
| --- | --- | --- | --- | --- | --- | --- | --- | --- | --- | --- | --- |
| 1 | 0.24 ± 0.11 | -0.8 ± 0.08 | 0.61 ± 0.03 | -0.22 ± 0.03 | 0.3 ± 0.03 |  |  | -0.54 ± 0.03 | 8080.51 | 0 | 0.73 |
| 2 | 0.24 ± 0.11 | -0.79 ± 0.08 | 0.6 ± 0.04 | -0.22 ± 0.03 | 0.3 ± 0.03 | -0.02 ± 0.03 |  | -0.54 ± 0.03 | 8083.98 | 3.46 | 0.13 |
| Averaged model | 0.24 ± 0.11 | -0.8 ± 0.79 | 0.61 ± 0.36 | -0.22 ± 0.08 | 0.3 ± 0.1 | -0.02 ± 0.03 | 0 ± 0 | -0.54 ± 0.03 |  |  |  |
