# Supplementary material for: Combining Methods to Describe Important Marine Habitats for Top Predators: Application to Identify Biological Hotspots in Tropical Waters
Source: PLoS One. 2014 Dec 10;9(12):e115057. doi: 10.1371/journal.pone.0115057 (PMC4262456; doi:10.1371/journal.pone.0115057)
Supplement: S4 Table — Ranked set of best candidates frigatebirds at-sea observations model and average model. Corrected Akaike Information Criterion (AICc), measure of each model AIC relative to the best one (d) and Akaike Weight (w) are presented. Values are mean ± SD. (DOC) [file pone.0115057.s006.doc]

| Model | INT | Chloa | SST | SLA | Bathy | DCol | | SST_  grad | SLA_  grad | Bathy_grad | AICc | d | w |
| --- | --- | --- | --- | --- | --- | --- | --- | --- | --- | --- | --- | --- | --- |
| 1 | -2.791 ± 0.34 | -0.59 ± 0.29 |  |  | 1.13 ± 0.30 | -2.77 ± 0.50 |  | |  |  | 237.1 | 0 | 0.2 |
| 2 | -2.627 ± 0.309 |  |  |  | 0.95 ± 0.28 | -2.65 ± 0.48 | |  |  |  | 238.11 | 1.01 | 0.12 |
| 3 | -2.823 ± 0.345 | -0.57 ± 0.29 |  | -0.26 ± 0.19 | 1.2 ± 0.31 | -2.83 ± 0.51 | |  |  |  | 239.15 | 2.05 | 0.07 |
| 4 | -2.863 ± 0.511 |  | 0.75 ± 0.51 |  | 1.05 ± 0.32 | -3.36 ± 0.67 | |  |  |  | 239.52 | 2.42 | 0.06 |
| 5 | -2.889 ± 0.356 | -0.66 ± 0.31 | 0.31 ± 0.24 |  | 1.20 ± 0.32 | -3.05 ± 0.57 | |  |  |  | 239.54 | 2.44 | 0.06 |
| 6 | -2.668 ± 0.316 |  |  | -0.29 ± 0.19 | 1.04 ± 0.3 | -2.73 ± 0.49 | |  |  |  | 239.79 | 2.69 | 0.05 |
| 7 | -2.949 ± 0.363 | -0.67 ± 0.31 | 0.4 ± 0.25 | -0.32 ± 0.19 | 1.31 ± 0.34 | -3.20 ± 0.59 | |  |  |  | 240.72 | 3.62 | 0.03 |
| 8 | -2.81 ± 0.35 | -0.57 ± 0.3 |  |  | 1.15 ± 0.31 | -2.8 ± 0.51 | | -0.15 ± 0.23 |  |  | 240.76 | 3.66 | 0.03 |
| 9 | -2.76 ± 0.33 |  |  | -0.4 ± 0.2 | 1.19 ± 0.32 | -2.89 ± 0.51 | | -0.40 ± 0.26 |  |  | 241.07 | 3.97 | 0.03 |
| 10 | -2.81 ± 0.35 | -0.56 ± 0.3 |  |  | 1.15 ± 0.31 | -2.82 ± 0.54 | |  | 0.06 ± 0.18 |  | 241.08 | 3.98 | 0.03 |
| 11 | -2.66 ± 0.32 |  |  |  | 1 ± 0.29 | -2.7 ± 0.49 | | -0.23 ± 0.22 |  |  | 241.1 | 4 | 0.03 |
| 12 | -2.78 ± 0.34 | -0.58 ± 0.29 |  |  | 1.09 ± 0.34 | -2.76 ± 0.50 | |  |  | 0.08 ± 0.27 | 241.11 | 4.01 | 0.03 |
| 13 | -2.7 ± 0.33 |  |  |  | 1.03 ± 0.3 | -2.82 ± 0.53 | |  | 0.16 ± 0.18 |  | 241.39 | 4.29 | 0.02 |
| 14 | -2.9 ± 0.5 |  | 0.81 ± 0.56 | -0.29 ± 0.2 | 1.12 ± 0.33 | -3.39 ± 0.68 | |  |  |  | 241.52 | 4.42 | 0.02 |
| 15 | -2.89 ± 0.36 | -0.52 ± 0.3 |  | -0.4 ± 0.2 | 1.29 ± 0.33 | -2.95 ± 0.53 | | -0.31 ± 0.26 |  |  | 241.72 | 4.62 | 0.02 |
| 16 | -2.62 ± 0.31 |  |  |  | 0.9 ± 0.31 | -2.65 ± 0.48 | |  |  | 0.12 ± 0.25 | 241.96 | 4.86 | 0.02 |
| 17 | -2.75 ± 0.34 |  |  | -0.29 ± 0.19 | 1.12 ± 0.32 | -2.92 ± 0.54 | |  | 0.17 ± 0.18 |  | 242.96 | 5.86 | 0.01 |
| 18 | -2.88 ± 0.49 |  | 0.63 ± 0.49 |  | 1.08 ± 0.32 | -3.36 ± 0.68 | | -0.18 ± 0.25 |  |  | 243.04 | 5.94 | 0.01 |
| 19 | -2.85 ± 0.35 | -0.53 ± 0.3 |  | -0.27 ± 0.19 | 1.24 ± 0.33 | -2.91 ± 0.55 | |  | 0.08 ± 0.18 |  | 243.07 | 5.97 | 0.01 |
| 20 | -2.81 ± 0.35 | -0.55 ± 0.29 |  | -0.26 ± 0.19 | 1.15 ± 0.35 | -2.83 ± 0.51 | |  |  | 0.09 ± 0.28 | 243.14 | 6.04 | 0.01 |
| 21 | -2.84 ± 0.51 |  | 0.77 ± 0.51 |  | 0.98 ± 0.34 | -3.35 ± 0.67 | |  |  | 0.15 ± 0.27 | 243.31 | 6.21 | 0.01 |
| 22 | -2.89 ± 0.36 | -0.64 ± 0.31 | 0.3 ± 0.24 |  | 1.21 ± 0.32 | -3.06 ± 0.57 | | -0.11 ± 0.23 |  |  | 243.42 | 6.32 | 0.01 |
| 23 | -2.88 ± 0.36 | -0.65 ± 0.31 | 0.33 ± 0.25 |  | 1.14 ± 0.36 | -3.06 ± 0.57 | |  |  | 0.12 ± 0.28 | 243.46 | 6.36 | 0.01 |
| 24 | -2.66 ± 0.32 |  |  | -0.29 ± 0.19 | 0.97 ± 0.32 | -2.73 ± 0.49 | |  |  | 0.14 ± 0.25 | 243.58 | 6.48 | 0.01 |
| 25 | -2.9 ± 0.36 | -0.64 ± 0.32 | 0.31 ± 0.24 |  | 1.22 ± 0.33 | -3.09 ± 0.6 | |  | 0.04 ± 0.19 |  | 243.59 | 6.49 | 0.01 |
| 26 | -2.86 ± 0.52 |  | 0.76 ± 0.56 |  | 1.05 ± 0.33 | -3.35 ± 0.67 | |  | -0.01 ± 0.20 |  | 243.6 | 6.5 | 0.01 |
| 27 | -2.98 ± 0.37 | -0.6 ± 0.32 | 0.37 ± 0.25 | -0.39 ± 0.2 | 1.38 ± 0.35 | -3.26 ± 0.59 | | -0.27 ± 0.26 |  |  | 243.7 | 6.61 | 0.01 |
| 28 | -2.94 ± 0.46 |  | 0.53 ± 0.56 | -0.41 ± 0.22 | 1.23 ± 0.34 | -3.39 ± 0.73 | | -0.37 ± 0.28 |  |  | 243.77 | 6.67 | 0.01 |
| 29 | -2.76 ± 0.34 |  |  | -0.43 ± 0.2 | 1.10 ± 0.33 | -2.91 ± 0.52 | | -0.44 ± 0.26 |  | 0.23 ± 0.25 | 244.38 | 7.28 | 0.01 |
| 30 | -2.83 ± 0.35 |  |  | -0.4 ± 0.2 | 1.25 ± 0.33 | -3.03 ± 0.56 | | -0.39 ± 0.26 | 0.14 ± 0.18 |  | 244.56 | 7.46 | 0.01 |
| 31 | -2.72 ± 0.33 |  |  |  | 1.06 ± 0.30 | -2.84 ± 0.53 | | -0.21 ± 0.23 | 0.14 ± 0.18 |  | 244.59 | 7.46 | 0.01 |
| 32 | -2.93 ± 0.36 | -0.66 ± 0.31 | 0.42 ± 0.26 | -0.33 ± 0.19 | 1.23 ± 0.37 | -3.21 ± 0.59 | |  |  | 0.14 ± 0.29 | 244.61 | 7.51 | 0.01 |
| 33 | -2.8 ± 0.35 | -0.55 ± 0.3 |  |  | 1.09 ± 0.34 | -2.79 ± 0.51 | | -0.17 ± 0.24 |  | 0.11 ± 0.27 | 244.69 | 7.59 | <0.01 |
| 34 | -2.97 ± 0.37 | -0.64 ± 0.32 | 0.4 ± 0.25 | -0.33 ± 0.19 | 1.34 ± 0.35 | -3.27 ± 0.62 | |  | 0.06 ± 0.19 |  | 244.72 | 7.62 | <0.01 |
| 35 | -2.65 ± 0.32 |  |  |  | 0.93 ± 0.31 | -2.70 ± 0.49 | | -0.25 ± 0.23 |  | 0.164 ± 0.244 | 244.75 | 7.65 | <0.01 |
| 36 | -2.82 ± 0.35 | -0.55 ± 0.31 |  |  | 1.17 ± 0.31 | -2.84 ± 0.54 | | -0.14 ± 0.23 | 0.05 ± 0.19 |  | 244.79 | 7.7 | <0.01 |
| Averaged model | -2.79 ± 0.36 | -0.59 ± 0.47 | 0.53 ± 0.28 | -0.31 ± 0.14 | 1.11 ± 1.20 | -2.89 ± 8.64 | | -0.26 ± 0.11 | 0.1 ± 0.03 | 0.117 ± 0.046 |  |  |  |
