# Supplementary material for: Combining Methods to Describe Important Marine Habitats for Top Predators: Application to Identify Biological Hotspots in Tropical Waters
Source: PLoS One. 2014 Dec 10;9(12):e115057. doi: 10.1371/journal.pone.0115057 (PMC4262456; doi:10.1371/journal.pone.0115057)
Supplement: S5 Table — Ranked set of best candidates frigatebirds at-sea observations model and average model. Corrected Akaike Information Criterion (AICc), measure of each model AIC relative to the best one (d) and Akaike Weight (w) are presented. Values are mean ± SD. (DOC) [file pone.0115057.s007.doc]

| Model | INT | Chloa | SST | SLA | Bathy | SST_grad | SLA_grad | Bathy_grad | AICc | d | w |
| --- | --- | --- | --- | --- | --- | --- | --- | --- | --- | --- | --- |
| 1 | -1.743 ± 0.347 | -0.585 ± 0.286 |  |  |  |  |  |  | 276.259 | 0 | 0.174 |
| 2 | -1.792 ± 0.327 | -0.733 ± 0.313 |  |  |  |  | -0.353 ± 0.193 |  | 276.402 | 0.143 | 0.162 |
| 3 | -1.649 ± 0.356 |  |  |  |  |  |  |  | 278.06 | 1.801 | 0.071 |
| 4 | -1.774 ± 0.568 | -0.662 ± 0.34 | 0.717 ± 0.523 |  |  |  | -0.431 ± 0.208 |  | 278.89 | 2.63 | 0.047 |
| 5 | -1.759 ± 0.351 | -0.623 ± 0.298 |  | -0.148 ± 0.166 |  |  |  |  | 279.518 | 3.259 | 0.034 |
| 6 | -1.659 ± 0.767 |  | 1.12 ± 0.471 |  |  |  | -0.401 ± 0.208 |  | 279.804 | 3.545 | 0.03 |
| 7 | -1.804 ± 0.33 | -0.756 ± 0.319 |  | -0.128 ± 0.174 |  |  | -0.347 ± 0.196 |  | 279.93 | 3.671 | 0.028 |
| 8 | -1.731 ± 0.446 | -0.554 ± 0.303 | 0.308 ± 0.494 |  |  |  |  |  | 279.944 | 3.685 | 0.028 |
| 9 | -1.746 ± 0.361 | -0.635 ± 0.3 |  |  | 0.112 ± 0.209 |  |  |  | 280.027 | 3.768 | 0.026 |
| 10 | -1.633 ± 0.611 |  | 0.691 ± 0.443 |  |  |  |  |  | 280.046 | 3.787 | 0.026 |
| 11 | -1.797 ± 0.319 | -0.722 ± 0.313 |  |  |  |  | -0.373 ± 0.198 | -0.096 ± 0.196 | 280.219 | 3.96 | 0.024 |
| 12 | -1.743 ± 0.346 | -0.594 ± 0.291 |  |  |  | 0.039 ± 0.192 |  |  | 280.271 | 4.012 | 0.023 |
| 13 | -1.743 ± 0.346 | -0.583 ± 0.289 |  |  |  |  |  | -0.007 ± 0.187 | 280.311 | 4.051 | 0.023 |
| 14 | -1.666 ± 0.345 |  |  |  |  |  | -0.228 ± 0.184 |  | 280.381 | 4.122 | 0.022 |
| 15 | -1.793 ± 0.332 | -0.751 ± 0.32 |  |  | 0.05 ± 0.205 |  | -0.348 ± 0.195 |  | 280.411 | 4.151 | 0.022 |
| 16 | -1.794 ± 0.328 | -0.73 ± 0.314 |  |  |  | -0.026 ± 0.204 | -0.357 ± 0.196 |  | 280.453 | 4.194 | 0.021 |
| 17 | -1.795 ± 0.625 | -0.7 ± 0.358 | 0.889 ± 0.525 | -0.213 ± 0.181 |  |  | -0.442 ± 0.212 |  | 281.602 | 5.343 | 0.012 |
| 18 | -1.657 ± 0.358 |  |  | -0.105 ± 0.165 |  |  |  |  | 281.692 | 5.433 | 0.011 |
| 19 | -1.651 ± 0.347 |  |  |  | -0.101 ± 0.178 |  |  |  | 281.773 | 5.513 | 0.011 |
| 20 | -1.652 ± 0.349 |  |  |  |  |  |  | -0.072 ± 0.183 | 281.939 | 5.68 | 0.01 |
| 21 | -1.649 ± 0.355 |  |  |  |  | -0.037 ± 0.188 |  |  | 282.06 | 5.801 | 0.01 |
| 22 | -1.777 ± 0.599 | -0.623 ± 0.343 | 0.813 ± 0.536 |  |  |  | -0.473 ± 0.217 | -0.143 ± 0.207 | 282.471 | 6.212 | 0.008 |
| 23 | -1.676 ± 0.823 |  | 1.267 ± 0.486 | -0.203 ± 0.175 |  |  | -0.413 ± 0.213 |  | 282.544 | 6.285 | 0.008 |
| 24 | -1.674 ± 0.804 |  | 1.242 ± 0.483 |  |  |  | -0.475 ± 0.22 | -0.22 ± 0.205 | 282.612 | 6.353 | 0.007 |
| 25 | -1.748 ± 0.512 | -0.592 ± 0.325 | 0.504 ± 0.504 | -0.202 ± 0.172 |  |  |  |  | 282.661 | 6.402 | 0.007 |
| 26 | -1.645 ± 0.673 |  | 0.854 ± 0.458 | -0.192 ± 0.167 |  |  |  |  | 282.809 | 6.55 | 0.007 |
| 27 | -1.775 ± 0.565 | -0.681 ± 0.354 | 0.701 ± 0.527 |  | 0.038 ± 0.217 |  | -0.426 ± 0.21 |  | 282.94 | 6.68 | 0.006 |
| 28 | -1.772 ± 0.574 | -0.665 ± 0.341 | 0.733 ± 0.531 |  |  | 0.028 ± 0.213 | -0.428 ± 0.209 |  | 282.952 | 6.693 | 0.006 |
| 29 | -1.768 ± 0.373 | -0.701 ± 0.317 |  | -0.175 ± 0.171 | 0.161 ± 0.219 |  |  |  | 283.04 | 6.781 | 0.006 |
| 30 | -1.667 ± 0.764 |  | 1.136 ± 0.478 |  | -0.159 ± 0.194 |  | -0.433 ± 0.21 |  | 283.184 | 6.925 | 0.005 |
| 31 | -1.759 ± 0.353 | -0.628 ± 0.303 |  | -0.151 ± 0.168 |  |  |  | 0.02 ± 0.19 | 283.573 | 7.314 | 0.004 |
| 32 | -1.759 ± 0.351 | -0.621 ± 0.3 |  | -0.149 ± 0.172 |  | -0.008 ± 0.204 |  |  | 283.583 | 7.324 | 0.004 |
| 33 | -1.67 ± 0.335 |  |  |  | -0.156 ± 0.179 |  | -0.259 ± 0.186 |  | 283.664 | 7.404 | 0.004 |
| 34 | -1.735 ± 0.458 | -0.61 ± 0.315 | 0.309 ± 0.475 |  | 0.121 ± 0.211 |  |  |  | 283.685 | 7.425 | 0.004 |
| 35 | -1.807 ± 0.341 | -0.791 ± 0.329 |  | -0.143 ± 0.178 | 0.086 ± 0.215 |  | -0.337 ± 0.198 |  | 283.848 | 7.589 | 0.004 |
| 36 | -1.807 ± 0.323 | -0.745 ± 0.319 |  | -0.116 ± 0.176 |  |  | -0.364 ± 0.201 | -0.075 ± 0.199 | 283.866 | 7.606 | 0.004 |
| 37 | -1.659 ± 0.768 |  | 1.121 ± 0.479 |  |  | 0.005 ± 0.211 | -0.401 ± 0.21 |  | 283.87 | 7.611 | 0.004 |
| 38 | -1.73 ± 0.465 | -0.566 ± 0.309 | 0.363 ± 0.512 |  |  | 0.076 ± 0.2 |  |  | 283.872 | 7.612 | 0.004 |
| 39 | -1.811 ± 0.334 | -0.751 ± 0.32 |  | -0.146 ± 0.182 |  | -0.075 ± 0.218 | -0.359 ± 0.2 |  | 283.887 | 7.628 | 0.004 |
| 40 | -1.672 ± 0.332 |  |  |  |  |  | -0.259 ± 0.19 | -0.136 ± 0.19 | 283.891 | 7.631 | 0.004 |
| 41 | -1.636 ± 0.609 |  | 0.7 ± 0.445 |  |  |  |  | -0.076 ± 0.186 | 283.924 | 7.664 | 0.004 |
| 42 | -1.636 ± 0.599 |  | 0.676 ± 0.449 |  | -0.067 ± 0.187 |  |  |  | 283.971 | 7.711 | 0.004 |
| 43 | -1.731 ± 0.446 | -0.552 ± 0.307 | 0.309 ± 0.496 |  |  |  |  | -0.009 ± 0.188 | 284.009 | 7.749 | 0.004 |
| 44 | -1.748 ± 0.359 | -0.634 ± 0.299 |  |  | 0.138 ± 0.229 |  |  | -0.059 ± 0.212 | 284.015 | 7.755 | 0.004 |
| 45 | -1.632 ± 0.626 |  | 0.727 ± 0.458 |  |  | 0.048 ± 0.198 |  |  | 284.043 | 7.783 | 0.004 |
| 46 | -1.746 ± 0.361 | -0.642 ± 0.304 |  |  | 0.111 ± 0.209 | 0.036 ± 0.193 |  |  | 284.059 | 7.799 | 0.004 |
| 47 | -1.801 ± 0.327 | -0.758 ± 0.319 |  |  | 0.106 ± 0.224 |  | -0.371 ± 0.199 | -0.137 ± 0.22 | 284.075 | 7.815 | 0.003 |
| 48 | -1.672 ± 0.348 |  |  | -0.093 ± 0.171 |  |  | -0.224 ± 0.186 |  | 284.142 | 7.883 | 0.003 |
| 49 | -1.668 ± 0.343 |  |  |  |  | -0.085 ± 0.196 | -0.243 ± 0.188 |  | 284.239 | 7.979 | 0.003 |
| 50 | -1.798 ± 0.32 | -0.721 ± 0.313 |  |  |  | -0.01 ± 0.207 | -0.375 ± 0.2 | -0.095 ± 0.198 | 284.297 | 8.038 | 0.003 |
| Averaged model | -1.738 ± 0.404 | -0.658 ± 0.528 | 0.758 ± 0.552 | -0.153 ± 0.054 | 0.043 ± 0.029 | 0.002 ± 0.032 | -0.367 ± 0.192 | -0.078 ± 0.038 |  |  |  |
