# Supplementary material for: Combining Methods to Describe Important Marine Habitats for Top Predators: Application to Identify Biological Hotspots in Tropical Waters
Source: PLoS One. 2014 Dec 10;9(12):e115057. doi: 10.1371/journal.pone.0115057 (PMC4262456; doi:10.1371/journal.pone.0115057)
Supplement: S7 Table — Ranked set of best candidates boobies at-sea observations model and average model. Corrected Akaike Information Criterion (AICc), measure of each model AIC relative to the best one (d) and Akaike Weight (w) are presented. Values are mean ± SD. (DOC) [file pone.0115057.s009.doc]

| Model | INT | Chloa | SST | SLA | Bathy | DCol | SST_  grad | SLA_grad | Bathy_  grad | AICc | d | w |
| --- | --- | --- | --- | --- | --- | --- | --- | --- | --- | --- | --- | --- |
| 1 | -2.27 ± 0.24 |  | 0.44 ± 0.2 |  |  | -1.17 ± 0.32 |  | -0.58 ± 0.25 |  | 235.94 | 0 | 0.1 |
| 2 | -2.18 ± 0.22 |  |  |  |  | -0.92 ± 0.26 |  | -0.5 ± 0.24 |  | 236.46 | 0.51 | 0.07 |
| 3 | -2.32 ± 0.25 |  | 0.45 ± 0.21 |  |  | -1.24 ± 0.34 | 0.277 ± 0.17 | -0.55 ± 0.25 |  | 237.53 | 1.59 | 0.04 |
| 4 | -2.22 ± 0.23 | 0.37 ± 0.17 |  |  |  | -1.26 ± 0.28 |  |  |  | 237.7 | 1.76 | 0.04 |
| 5 | -2.31 ± 0.25 | 0.3 ± 0.19 | 0.41 ± 0.2 |  |  | -1.3 ± 0.33 |  | -0.52 ± 0.26 |  | 237.74 | 1.8 | 0.04 |
| 6 | -2.23 ± 0.23 | 0.31 ± 0.18 |  |  |  | -1.06 ± 0.28 |  | -0.45 ± 0.25 |  | 237.85 | 1.9 | 0.04 |
| 7 | -2.16 ± 0.23 |  |  |  |  | -1.09 ± 0.27 |  |  |  | 237.89 | 1.94 | 0.04 |
| 8 | -2.21 ± 0.23 |  |  |  |  | -0.95 ± 0.26 | 0.28 ± 0.17 | -0.48 ± 0.24 |  | 238.1 | 2.16 | 0.03 |
| 9 | -2.31 ± 0.25 |  | 0.47 ± 0.21 |  |  | -1.38 ± 0.37 |  | -0.51 ± 0.26 | 0.29 ± 0.22 | 238.33 | 2.39 | 0.03 |
| 10 | -2.27 ± 0.25 |  | 0.4 ± 0.21 |  |  | -1.4 ± 0.34 |  |  |  | 238.36 | 2.42 | 0.03 |
| 11 | -2.36 ± 0.26 |  | 0.43 ± 0.2 |  | 0.33 ± 0.27 | -1.47 ± 0.43 |  | -0.55 ± 0.26 |  | 238.46 | 2.52 | 0.03 |
| 12 | -2.32 ± 0.25 | 0.38 ± 0.18 | 0.38 ± 0.21 |  |  | -1.54 ± 0.35 |  |  |  | 238.47 | 2.53 | 0.03 |
| 13 | -2.21 ± 0.23 |  |  |  |  | -1.14 ± 0.27 | 0.309 ± 0.165 |  |  | 238.78 | 2.83 | 0.02 |
| 14 | -2.26 ± 0.24 |  |  |  | 0.31 ± 0.25 | -1.2 ± 0.37 |  | -0.48 ± 0.24 |  | 238.87 | 2.93 | 0.02 |
| 15 | -2.35 ± 0.26 |  | 0.47 ± 0.22 |  |  | -1.67 ± 0.39 |  |  | 0.4 ± 0.21 | 239 | 3.06 | 0.02 |
| 16 | -2.34 ± 0.26 |  | 0.41 ± 0.22 |  |  | -1.49 ± 0.36 | 0.309 ± 0.162 |  |  | 239.13 | 3.19 | 0.02 |
| 17 | -2.22 ± 0.28 |  |  |  |  | -1.28 ± 0.32 |  |  | 0.37 ± 0.21 | 239.14 | 3.19 | 0.02 |
| 18 | -2.2 ± 0.22 |  |  |  |  | -1.05 ± 0.29 |  | -0.46 ± 0.24 | 0.24 ± 0.21 | 239.23 | 3.29 | 0.02 |
| 19 | -2.3 ± 0.29 |  |  |  | 0.43 ± 0.27 | -1.48 ± 0.4 |  |  |  | 239.25 | 3.31 | 0.02 |
| 20 | -2.4 ± 0.28 |  | 0.4 ± 0.21 |  | 0.44 ± 0.28 | -1.81 ± 0.45 |  |  |  | 239.84 | 3.9 | 0.01 |
| 21 | -2.25 ± 0.23 | 0.34 ± 0.17 |  |  |  | -1.39 ± 0.31 |  |  | 0.29 ± 0.21 | 239.96 | 4.01 | 0.01 |
| 22 | -2.27 ± 0.24 |  | 0.44 ± 0.21 | -0.04 ± 0.2 |  | -1.18 ± 0.32 |  | -0.57 ± 0.25 |  | 239.99 | 4.05 | 0.01 |
| 23 | -2.25 ± 0.24 | 0.31 ± 0.18 |  |  |  | -1.27 ± 0.29 | 0.235 ± 0.175 |  |  | 240.09 | 4.15 | 0.01 |
| 24 | -2.38 ± 0.26 | 0.33 ± 0.18 | 0.42 ± 0.22 |  |  | -1.75 ± 0.39 |  |  | 0.34 ± 0.22 | 240.2 | 4.26 | 0.01 |
| 25 | -2.35 ± 0.25 | 0.23 ± 0.2 | 0.42 ± 0.21 |  |  | -1.33 ± 0.35 | 0.224 ± 0.177 | -0.51 ± 0.26 |  | 240.33 | 4.39 | 0.01 |
| 26 | -2.25 ± 0.23 | 0.25 ± 0.18 |  |  |  | -1.07 ± 0.29 | 0.225 ± 0.178 | -0.44 ± 0.25 |  | 240.44 | 4.49 | 0.01 |
| 27 | -2.18 ± 0.22 |  |  | 0.04 ± 0.19 |  | -0.92 ± 0.26 |  | -0.5 ± 0.24 |  | 240.49 | 4.55 | 0.01 |
| 28 | -2.35 ± 0.25 | 0.27 ± 0.19 | 0.44 ± 0.21 |  |  | -1.46 ± 0.38 |  | -0.47 ± 0.26 | 0.25 ± 0.22 | 240.55 | 4.61 | 0.01 |
| 29 | -2.35 ± 0.26 |  | 0.47 ± 0.21 |  |  | -1.39 ± 0.39 | 0.233 ± 0.17 | -0.5 ± 0.26 | 0.22 ± 0.23 | 240.72 | 4.78 | 0.01 |
| 30 | -2.39 ± 0.27 |  | 0.44 ± 0.21 |  | 0.26 ± 0.28 | -1.48 ± 0.44 | 0.24 ± 0.173 | -0.52 ± 0.26 |  | 240.73 | 4.79 | 0.01 |
| 31 | -2.37 ± 0.26 | 0.31 ± 0.19 | 0.39 ± 0.21 |  |  | -1.58 ± 0.36 | 0.235 ± 0.173 |  |  | 240.84 | 4.9 | 0.01 |
| 32 | -2.24 ± 0.23 | 0.29 ± 0.18 |  |  |  | -1.17 ± 0.31 |  | -0.41 ± 0.25 | 0.22 ± 0.21 | 240.87 | 4.92 | 0.01 |
| 33 | -2.29 ± 0.25 | 0.3 ± 0.18 |  |  | 0.26 ± 0.28 | -1.47 ± 0.38 |  |  |  | 240.89 | 4.94 | 0.01 |
| 34 | -2.28 ± 0.25 |  |  |  | 0.255 ± 0.25 | -1.19 ± 0.38 | 0.246 ± 0.176 | -0.46 ± 0.24 |  | 241.14 | 5.2 | 0.01 |
| 35 | -2.31 ± 0.27 |  |  |  | 0.35 ± 0.28 | -1.46 ± 0.4 | 0.264 ± 0.174 |  |  | 241.17 | 5.23 | 0.01 |
| 36 | -2.39 ± 0.27 |  | 0.46 ± 0.22 |  |  | -1.7 ± 0.4 | 0.24 ± 0.169 |  | 0.33 ± 0.22 | 241.18 | 5.24 | 0.01 |
| 37 | -2.37 ± 0.27 | 0.24 ± 0.2 | 0.41 ± 0.2 |  | 0.23 ± 0.28 | -1.48 ± 0.43 |  | -0.51 ± 0.26 |  | 241.19 | 5.25 | 0.01 |
| 38 | -2.28 ± 0.25 | 0.25 ± 0.19 |  |  | 0.21 ± 0.26 | -1.24 ± 0.4 |  | -0.44 ± 0.25 |  | 241.28 | 5.33 | 0.01 |
| 39 | -2.24 ± 0.26 |  |  |  |  | -1.28 ± 0.31 | 0.25 ± 0.18 |  | 0.29 ± 0.23 | 241.34 | 5.39 | 0.01 |
| 40 | -2.23 ± 0.23 |  |  |  |  | -1.05 ± 0.3 | 0.25 ± 0.18 | -0.45 ± 0.24 | 0.19 ± 0.22 | 241.49 | 5.55 | 0.01 |
| 41 | -2.44 ± 0.28 |  | 0.41 ± 0.22 |  | 0.37 ± 0.29 | -1.81 ± 0.46 | 0.27 ± 0.17 |  |  | 241.59 | 5.64 | 0.01 |
| 42 | -2.4 ± 0.28 | 0.3 ± 0.19 | 0.38 ± 0.21 |  | 0.29 ± 0.3 | -1.78 ± 0.45 |  |  |  | 241.62 | 5.67 | 0.01 |
| 43 | -2.32 ± 0.25 |  | 0.44 ± 0.21 | 0.02 ± 0.21 |  | -1.24 ± 0.34 | 0.28 ± 0.17 | -0.55 ± 0.25 |  | 241.62 | 5.68 | 0.01 |
| 44 | -2.22 ± 0.23 | 0.37 ± 0.17 |  | 0.02 ± 0.19 |  | -1.26 ± 0.28 |  |  |  | 241.77 | 5.82 | 0.01 |
| 45 | -2.31 ± 0.25 | 0.3 ± 0.19 | 0.42 ± 0.2 | -0.04 ± 0.21 |  | -1.3 ± 0.33 |  | -0.52 ± 0.26 |  | 241.79 | 5.85 | 0.01 |
| 46 | -2.36 ± 0.27 |  | 0.45 ± 0.21 |  | 0.23 ± 0.29 | -1.53 ± 0.44 |  | -0.51 ± 0.26 | 0.21 ± 0.24 | 241.82 | 5.87 | 0.01 |
| 47 | -2.16 ± 0.23 |  |  | 0.04 ± 0.19 |  | -1.09 ± 0.27 |  |  |  | 241.91 | 5.96 | 0.01 |
| 48 | -2.23 ± 0.23 | 0.31 ± 0.18 |  | 0.02 ± 0.2 |  | -1.06 ± 0.28 |  | -0.45 ± 0.25 |  | 241.92 | 5.97 | 0.01 |
| 49 | -2.22 ± 0.23 |  |  | 0.1 ± 0.2 |  | -0.96 ± 0.27 | 0.3 ± 0.17 | -0.48 ± 0.24 |  | 241.94 | 5.99 | 0.01 |
| 50 | -2.31 ± 0.31 |  |  |  | 0.31 ± 0.29 | -1.51 ± 0.41 |  |  | 0.28 ± 0.24 | 242.05 | 6.1 | 0.01 |
| 51 | -2.41 ± 0.28 |  | 0.45 ± 0.22 |  | 0.27 ± 0.31 | -1.86 ± 0.46 |  |  | 0.32 ± 0.24 | 242.32 | 6.37 | <0.01 |
| 52 | -2.31 ± 0.25 |  | 0.48 ± 0.22 | -0.06 ± 0.2 |  | -1.38 ± 0.38 |  | -0.51 ± 0.26 | 0.29 ± 0.22 | 242.33 | 6.39 | <0.01 |
| 53 | -2.27 ± 0.25 |  | 0.41 ± 0.22 | -0.05 ± 0.2 |  | -1.41 ± 0.34 |  |  |  | 242.36 | 6.42 | <0.01 |
| 54 | -2.26 ± 0.26 |  |  |  | 0.25 ± 0.27 | -1.23 ± 0.38 |  | -0.45 ± 0.25 | 0.17 ± 0.24 | 242.42 | 6.48 | <0.01 |
| 55 | -2.32 ± 0.25 | 0.38 ± 0.18 | 0.39 ± 0.21 | -0.06 ± 0.2 |  | -1.54 ± 0.35 |  |  |  | 242.47 | 6.53 | <0.01 |
| 56 | -2.36 ± 0.26 |  | 0.44 ± 0.21 | -0.06 ± 0.2 | 0.34 ± 0.27 | -1.48 ± 0.44 |  | -0.54 ± 0.26 |  | 242.48 | 6.54 | <0.01 |
| 57 | -2.22 ± 0.23 |  |  | 0.09 ± 0.19 |  | -1.15 ± 0.27 | 0.32 ± 0.17 |  |  | 242.63 | 6.69 | <0.01 |
| 58 | -2.35 ± 0.26 |  | 0.49 ± 0.23 | -0.08 ± 0.2 |  | -1.69 ± 0.39 |  |  | 0.41 ± 0.21 | 242.9 | 6.96 | <0.01 |
| 59 | -2.26 ± 0.24 |  |  | 0.01 ± 0.19 | 0.31 ± 0.25 | -1.2 ± 0.37 |  | -0.48 ± 0.24 |  | 242.95 | 7 | <0.01 |
| 60 | -2.27 ± 0.24 | 0.29 ± 0.18 |  |  |  | -1.38 ± 0.31 | 0.19 ± 0.18 |  | 0.25 ± 0.22 | 243.01 | 7.07 | <0.01 |
| 61 | -2.22 ± 0.28 |  |  | -0.01 ± 0.2 |  | -1.28 ± 0.32 |  |  | 0.37 ± 0.22 | 243.21 | 7.26 | <0.01 |
| 62 | -2.34 ± 0.26 |  | 0.42 ± 0.22 | -0.01 ± 0.2 |  | -1.49 ± 0.36 | 0.31 ± 0.16 |  |  | 243.21 | 7.27 | <0.01 |
| 63 | -2.31 ± 0.3 |  |  | -0.03 ± 0.2 | 0.44 ± 0.27 | -1.48 ± 0.41 |  |  |  | 243.3 | 7.36 | <0.01 |
| 64 | -2.2 ± 0.22 |  |  | 0.01 ± 0.2 |  | -1.05 ± 0.29 |  | -0.46 ± 0.24 | 0.24 ± 0.21 | 243.31 | 7.36 | <0.01 |
| 65 | -2.41 ± 0.27 | 0.28 ± 0.19 | 0.43 ± 0.22 |  |  | -1.76 ± 0.4 | 0.18 ± 0.18 |  | 0.3 ± 0.23 | 243.36 | 7.42 | <0.01 |
| 66 | -2.31 ± 0.26 | 0.25 ± 0.19 |  |  | 0.22 ± 0.28 | -1.45 ± 0.39 | 0.22 ± 0.18 |  |  | 243.55 | 7.61 | <0.01 |
| Averaged model | -2.27 ± 0.24 | 0.32 ± 0.11 | 0.43 ± 0.19 | -0.01 ± 0.01 | 0.32 ± 0.11 | -1.27 ± 1.65 | 0.27 ± 0.08 | -0.51 ± 0.3 | 0.3 ± 0.1 |  |  |  |
