# Supplementary material for: Combining Methods to Describe Important Marine Habitats for Top Predators: Application to Identify Biological Hotspots in Tropical Waters
Source: PLoS One. 2014 Dec 10;9(12):e115057. doi: 10.1371/journal.pone.0115057 (PMC4262456; doi:10.1371/journal.pone.0115057)
Supplement: S8 Table — Ranked set of best candidates sub-surface predators at-sea observations model and average model. Corrected Akaike Information Criterion (AICc), measure of each model AIC relative to the best one (d) and Akaike Weight (w) are presented. Values are mean ± SD. (DOC) [file pone.0115057.s010.doc]

| Model | INT | SST | SLA | Bathy | Chloa_  grad | SST_  grad | SLA_  grad | Bathy_  grad | AICc | d | w |
| --- | --- | --- | --- | --- | --- | --- | --- | --- | --- | --- | --- |
| 1 | -1.86 ± 0.18 | 0.56 ± 0.16 |  |  |  |  | -0.56 ± 0.23 | -0.42 ± 0.2 | 268.98 | 0 | 0.19 |
| 2 | -1.81 ± 0.17 | 0.5 ± 0.16 |  |  |  |  | -0.45 ± 0.22 |  | 270.26 | 1.29 | 0.10 |
| 3 | -1.56 ± 0.17 | 0.42 ± 0.15 |  |  |  |  |  |  | 271.15 | 2.17 | 0.06 |
| 4 | -1.78 ± 0.17 | 0.45 ± 0.15 |  |  |  |  |  | -0.32 ± 0.19 | 272.1 | 3.12 | 0.04 |
| 5 | -1.83 ± 0.18 | 0.53 ± 0.16 |  |  |  | -0.25 ± 0.18 | -0.48 ± 0.22 |  | 272.11 | 3.13 | 0.04 |
| 6 | -1.87 ± 0.18 | 0.59 ± 0.16 |  |  | 0.16 ± 0.16 |  | -0.57 ± 0.23 | -0.47 ± 0.21 | 272.21 | 3.23 | 0.04 |
| 7 | -1.86 ± 0.18 | 0.56 ± 0.16 |  |  |  | -0.14 ± 0.19 | -0.56 ± 0.23 | -0.37 ± 0.21 | 272.51 | 3.53 | 0.03 |
| 8 | -1.87 ± 0.31 |  |  |  |  |  | -0.34 ± 0.22 |  | 272.63 | 3.65 | 0.03 |
| 9 | -1.87 ± 0.18 | 0.56 ± 0.16 |  | 0.13 ± 0.2 |  |  | -0.56 ± 0.23 | -0.5 ± 0.24 | 272.64 | 3.66 | 0.03 |
| 10 | -1.93 ± 0.33 |  |  |  |  |  | -0.43 ± 0.23 | -0.37 ± 0.2 | 272.76 | 3.78 | 0.03 |
| 11 | -1.86 ± 0.18 | 0.58 ± 0.16 | -0.1 ± 0.18 |  |  |  | -0.55 ± 0.23 | -0.42 ± 0.2 | 272.77 | 3.79 | 0.03 |
| 12 | -1.86 ± 0.3 |  |  |  |  |  |  | -0.28 ± 0.19 | 273.02 | 4.04 | 0.03 |
| 13 | -1.85 ± 0.31 |  |  |  |  | -0.24 ± 0.18 |  |  | 273.38 | 4.4 | 0.02 |
| 14 | -1.77 ± 0.17 | 0.44 ± 0.15 |  |  |  | -0.2 ± 0.17 |  |  | 273.69 | 4.71 | 0.02 |
| 15 | -1.91 ± 0.33 |  |  |  |  | -0.3 ± 0.19 | -0.4 ± 0.223 |  | 273.76 | 4.78 | 0.02 |
| 16 | -1.81 ± 0.17 | 0.52 ± 0.16 |  | -0.1 ± 0.17 |  |  | -0.46 ± 0.22 |  | 273.97 | 4.99 | 0.02 |
| 17 | -1.81 ± 0.17 | 0.52 ± 0.16 | -0.1 ± 0.17 |  |  |  | -0.44 ± 0.22 |  | 274 | 5.02 | 0.02 |
| 18 | -1.81 ± 0.17 | 0.51 ± 0.16 |  |  | 0.06 ± 0.16 |  | -0.45 ± 0.22 |  | 274.2 | 5.22 | 0.01 |
| 19 | -1.76 ± 0.17 | 0.45 ± 0.15 | -0.11 ± 0.16 |  |  |  |  |  | 274.73 | 5.75 | 0.01 |
| 20 | -1.76 ± 0.17 | 0.43 ± 0.15 |  |  | 0.06 ± 0.16 |  |  |  | 275.06 | 6.08 | 0.01 |
| 21 | -1.76 ± 0.17 | 0.43 ± 0.15 |  | -0.06 ± 0.17 |  |  |  |  | 275.09 | 6.11 | 0.01 |
| 22 | -1.83 ± 0.3 |  | -0.09 ± 0.17 |  |  |  |  |  | 275.17 | 6.19 | 0.01 |
| 23 | -1.82 ± 0.3 |  |  |  | 0.04 ± 0.16 |  |  |  | 275.37 | 6.39 | 0.01 |
| 24 | -1.84 ± 0.18 | 0.55 ± 0.16 | -0.15 ± 0.17 |  |  | -0.29 ± 0.19 | -0.48 ± 0.22 |  | 275.41 | 6.43 | 0.01 |
| 25 | -1.82 ± 0.3 |  |  | 0.01 ± 0.18 |  |  |  |  | 275.44 | 6.46 | 0.01 |
| 26 | -1.79 ± 0.17 | 0.47 ± 0.15 |  |  | 0.14 ± 0.16 |  |  | -0.36 ± 0.21 | 275.47 | 6.49 | 0.01 |
| 27 | -1.94 ± 0.33 |  |  |  |  | -0.21 ± 0.2 | -0.46 ± 0.23 | -0.29 ± 0.21 | 275.62 | 6.64 | 0.01 |
| 28 | -1.79 ± 0.17 | 0.44 ± 0.15 |  | 0.15 ± 0.2 |  |  |  | -0.41 ± 0.24 | 275.67 | 6.69 | 0.01 |
| 29 | -1.87 ± 0.18 | 0.59 ± 0.16 |  |  | 0.16 ± 0.15 | -0.15 ± 0.19 | -0.58 ± 0.23 | -0.41 ± 0.22 | 275.7 | 6.72 | 0.01 |
| 30 | -1.79 ± 0.17 | 0.47 ± 0.16 | -0.11 ± 0.16 |  |  |  |  | -0.32 ± 0.19 | 275.73 | 6.75 | 0.01 |
| 31 | -1.88 ± 0.18 | 0.62 ± 0.17 | -0.13 ± 0.18 |  | 0.19 ± 0.16 |  | -0.57 ± 0.23 | -0.47 ± 0.21 | 275.75 | 6.78 | 0.01 |
| 32 | -1.79 ± 0.17 | 0.45 ± 0.15 |  |  |  | -0.11 ± 0.18 |  | -0.27 ± 0.2 | 275.76 | 6.78 | 0.01 |
| 33 | -1.83 ± 0.18 | 0.54 ± 0.16 |  |  | 0.09 ± 0.15 | -0.27 ± 0.18 | -0.48 ± 0.23 |  | 275.86 | 6.88 | 0.01 |
| 34 | -1.96 ± 0.35 |  |  | 0.2 ± 0.21 |  |  | -0.43 ± 0.23 | -0.48 ± 0.25 | 275.97 | 6.99 | 0.01 |
| 35 | -1.88 ± 0.32 |  |  | 0.22 ± 0.22 |  |  |  | -0.42 ± 0.25 | 275.98 | 7.01 | 0.01 |
| 36 | -1.87 ± 0.18 | 0.55 ± 0.16 |  | 0.15 ± 0.2 |  | -0.16 ± 0.2 | -0.57 ± 0.23 | -0.45 ± 0.24 | 276.06 | 7.08 | 0.01 |
| 37 | -1.87 ± 0.18 | 0.58 ± 0.16 | -0.13 ± 0.18 |  |  | -0.17 ± 0.2 | -0.56 ± 0.23 | -0.36 ± 0.21 | 276.11 | 7.13 | 0.01 |
| 38 | -1.83 ± 0.18 | 0.53 ± 0.16 |  | -0.03 ± 0.18 |  | -0.25 ± 0.19 | -0.48 ± 0.22 |  | 276.16 | 7.18 | 0.01 |
| 39 | -1.86 ± 0.31 |  |  |  |  | -0.17 ± 0.19 |  | -0.21 ± 0.2 | 276.22 | 7.24 | 0.01 |
| 40 | -1.87 ± 0.18 | 0.58 ± 0.16 |  | 0.05 ± 0.23 | 0.13 ± 0.18 |  | -0.57 ± 0.23 | -0.49 ± 0.24 | 276.25 | 7.27 | 0.01 |
| 41 | -1.87 ± 0.18 | 0.57 ± 0.16 | -0.1 ± 0.18 | 0.14 ± 0.2 |  |  | -0.55 ± 0.23 | -0.5 ± 0.24 | 276.39 | 7.41 | 0.01 |
| 42 | -1.94 ± 0.33 |  |  |  | 0.1 ± 0.16 |  | -0.43 ± 0.23 | -0.4 ± 0.21 | 276.45 | 7.47 | <0.01 |
| 43 | -1.88 ± 0.32 |  | -0.09 ± 0.18 |  |  |  | -0.34 ± 0.22 |  | 276.46 | 7.48 | <0.01 |
| 44 | -1.88 ± 0.33 |  | -0.17 ± 0.18 |  |  | -0.29 ± 0.19 |  |  | 276.54 | 7.56 | <0.01 |
| 45 | -1.87 ± 0.31 |  |  | -0.03 ± 0.18 |  |  | -0.34 ± 0.22 |  | 276.64 | 7.67 | <0.01 |
| 46 | -1.87 ± 0.31 |  |  |  | 0.03 ± 0.17 |  | -0.34 ± 0.22 |  | 276.65 | 7.67 | <0.01 |
| 47 | -1.86 ± 0.31 |  |  |  | 0.11 ± 0.16 |  |  | -0.31 ± 0.2 | 276.66 | 7.68 | <0.01 |
| 48 | -1.94 ± 0.34 |  | -0.07 ± 0.18 |  |  |  | -0.43 ± 0.23 | -0.37 ± 0.2 | 276.68 | 7.7 | <0.01 |
| 49 | -1.95 ± 0.36 |  | -0.2 ± 0.19 |  |  | -0.36 ± 0.2 | -0.42 ± 0.23 |  | 276.76 | 7.78 | <0.01 |
| 50 | -1.86 ± 0.31 |  | -0.08 ± 0.17 |  |  |  |  | -0.28 ± 0.2 | 276.88 | 7.9 | <0.01 |
| 51 | -1.78 ± 0.17 | 0.47 ± 0.157 | -0.15 ± 0.16 |  |  | -0.23 ± 0.18 |  |  | 276.9 | 7.92 | <0.01 |
| 52 | -1.85 ± 0.32 |  |  |  | 0.08 ± 0.16 | -0.26 ± 0.18 |  |  | 277.17 | 8.19 | <0.01 |
| 53 | -1.86 ± 0.32 |  |  | 0.08 ± 0.19 |  | -0.26 ± 0.19 |  |  | 277.22 | 8.25 | <0.01 |
| 54 | -1.82 ± 0.18 | 0.55 ± 0.17 |  | -0.19 ± 0.2 | 0.16 ± 0.19 |  | -0.47 ± 0.22 |  | 277.41 | 8.43 | <0.01 |
| 55 | -1.77 ± 0.17 | 0.45 ± 0.16 |  |  | 0.09 ± 0.15 | -0.21 ± 0.17 |  |  | 277.42 | 8.44 | <0.01 |
| 56 | -1.92 ± 0.33 |  |  |  | 0.07 ± 0.16 | -0.31 ± 0.19 | -0.4 ± 0.22 |  | 277.64 | 8.66 | <0.01 |
| 57 | -1.77 ± 0.17 | 0.44 ± 0.15 |  | 0.01 ± 0.18 |  | -0.2 ± 0.18 |  |  | 277.75 | 8.77 | <0.01 |
| Averaged model | -1.84 ± 0.21 | 0.52 ± 0.27 | -0.11 ± 0.04 | 0.06 ± 0.03 | 0.12 ± 0.03 | -0.22 ± 0.08 | -0.5 ± 0.31 | -0.4 ± 0.22 |  |  |  |
